# Development of a Standard Tool of Pattern Identification for Functional Dyspepsia: A Cross-Sectional Study from Korea

**DOI:** 10.3390/healthcare12232331

**Published:** 2024-11-21

**Authors:** Na-Yeon Ha, Seok-Jae Ko, Jae-Woo Park, Jinsung Kim

**Affiliations:** 1 Department of Digestive Diseases, College of Korean Medicine, Kyung Hee University, 26 Kyungheedae-ro, Dongdaemun-gu, Seoul 02447, Republic of Korea; 2 Division of Digestive Diseases, Department of Korean Internal Medicine, Kyung Hee University Korean Medicine Hospital, 23 Kyungheedae-ro, Dongdaemun-gu, Seoul 02447, Republic of Korea; 3 Department of Internal Korean Medicine, Kyung Hee University Hospital at Gangdong, 892 Dongnam-ro, Gangdong-gu, Seoul 05278, Republic of Korea

**Keywords:** functional dyspepsia, pattern identification, syndrome differentiation, questionnaire

## Abstract

Background/objective: The diagnosis and treatment of functional dyspepsia (FD) require a systematic and tailored approach. In traditional Korean medicine (TKM), pattern-identification tools help analyze clinical information and guide treatment. This study aimed to develop a Korean version of the standard tool of pattern identification for functional dyspepsia (STPI-FD) and subsequently assess its reliability and validity. Methods: Common patterns and symptoms were identified through a systematic review of the clinical studies conducted in Korea and China. An importance survey for each pattern and symptom was conducted using the Delphi method and refined through expert consensus. A draft STPI-FD comprising six patterns and 38 items was developed. Ninety-five patients with FD completed the STPI-FD along with the Visual Analog Scale, Total Dyspepsia Symptom scale, Single Dyspepsia Symptom scale, and Functional Dyspepsia-related Quality of Life questionnaire. Results: Two items were excluded from the STPI-FD due to their negative impact on reliability, resulting in a 36-item tool. The revised STPI-FD demonstrated high internal consistency, with an overall Cronbach’s alpha of 0.942. In contrast, the Cronbach’s alpha values for each pattern ranged from 0.7 to 0.9. To assess its validity, significant differences in the pattern characteristics and associated symptoms were confirmed, indicating its clinical relevance. Statistically significant positive correlations between the revised STPI-FD and other dyspepsia-related scales underscore the differentiation between patterns. Conclusion: The final STPI-FD is a moderately reliable and valid tool for diagnosing specific patterns in FD, supporting the selection of interventions and the evaluation of symptom improvement in patients treated with TKM.

## 1. Introduction

Functional dyspepsia (FD) is a gastrointestinal disorder characterized by several chronic symptoms, including epigastric pain, burning, postprandial fullness, and early satiation, without any identifiable organic cause or systemic disease [1]. The prevalence of FD is high, affecting approximately 10–30 of every 100 individuals [2], and it is associated with reduced quality of life and psychosocial impairments, such as anxiety and depression [3]. This is also linked to socioeconomic losses owing to decreased productivity [4]. The pathophysiological mechanisms underlying FD are complex and poorly understood, although they are known to involve delayed gastric emptying [5], impaired gastric accommodation [6], hypersensitivity to gastric distention [7], and altered mechanisms in the brain–gut axis [8]. Therefore, symptomatic treatment with medications, such as proton pump inhibitors, histamine-type-2-receptor antagonists, prokinetic agents, and tricyclic antidepressants, is commonly employed in clinical practice. However, alternative therapies, such as herbal therapies, have also emerged as potential therapeutic options [9].

In clinical practice, dyspepsia symptoms are often expressed in ambiguous terms, leading to a variety of descriptions and frequent communication issues between patients and physicians [1]. To address this issue, the Rome criteria have established representative diagnostic standards for FD. The most recent version of these guidelines, the Rome IV criteria, was revised in 2016 [10], and is currently used in clinical settings. According to the Rome criteria, FD is broadly classified based on whether the symptoms are triggered by food intake. These can be divided into postprandial distress syndrome (PDS), epigastric pain syndrome (EPS), and overlapping categories.

Pattern identification (or syndrome differentiation) is a unique clinical diagnostic process commonly used in traditional complementary or alternative medical systems in Asian countries. This process involves comprehensively collecting, analyzing, and subdividing the complete information of a patient gathered through inspection, listening, smell, inquiry, and palpation to determine the location, cause, and nature of the disease. This information can subsequently be used to prescribe herbal medicines and to evaluate treatment effectiveness and prognosis [11]. However, in the absence of specific guidelines for pattern identification of each symptom or disease, pattern identification tends to rely on the subjective opinions and experiences of individual practitioners. This can lead to issues with reproducibility, reliability, and objectivity. Consequently, there has been a recent surge in the development of questionnaires and diagnostic tools aimed at standardizing pattern identification. Questionnaires based on traditional Korean medicine (TKM) diagnoses, such as the cold–heat pattern [12], deficiency–excess pattern [13], blood stasis pattern [14], and phlegm-retained fluid pattern [15], have also been developed. Pattern-identification tools for dyspepsia have specifically been developed to differentiate food retention disorder (FRDO) [16], spleen qi deficiency pattern [17], and stomach qi deficiency pattern [18]. One study on the frequency of pattern identification among 97 Korean patients with FD used a pattern identification instrument based on the Chinese clinical guidelines for FD [19]. The distributions of the five identified patterns were as follows: spleen deficiency and phlegm dampness; liver depression and spleen deficiency; liver depression and qi stagnation; tangled cold and heat (TACH); and food accumulation [20]. In 2010, a pattern-identification tool for FD was developed in Korea. This tool comprises 45 items, 12 of which are related to tongue and pulse diagnoses, and categorizes FD symptoms into six types: liver–stomach disharmony (LSDH); dampness and heat in the spleen and stomach (DHSS); spleen and stomach deficiency and cold (SSDC); TACH; food retention disorder; and insufficiency of stomach yin [21]. However, because the symptom items and indicators for pattern identification were extracted solely from classical textbooks, their reliability and validity have not yet been tested in patients. Furthermore, opinions regarding the clinical application of certain patterns differ and modifications and improvements are required before practical clinical applications can be implemented.

In this context, the standardization and simplification of pattern identification based on symptoms are essential to establish clinical evidence and indicators for evaluating the effectiveness of diagnosis and treatment in TKM. In response to the increasing need for pattern-identification questionnaires, we aimed to develop a contemporary pattern-identification tool for clinically significant patterns frequently diagnosed in patients with FD.

## 2. Materials and Methods

**Figure 1:**
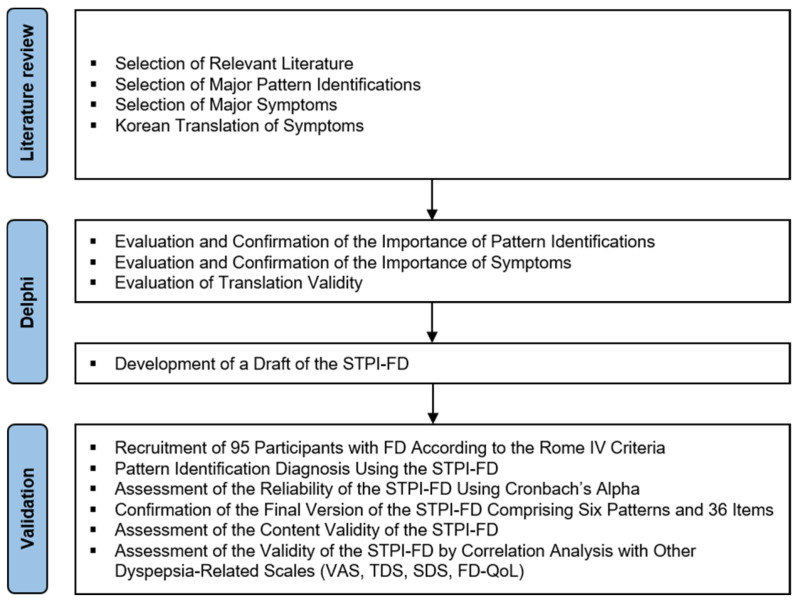
Development process of the STPI-FD. Abbreviations: FD-QoL, functional dyspepsia-related quality of life; SDS, single dyspepsia symptoms; STPI-FD, standard tool of pattern identification for functional dyspepsia; TDS, total dyspepsia symptoms; VAS, visual analog scale.

To supplement the previously developed pattern identification questionnaire for FD, we reviewed recent clinical trial papers as reference literature, seeking consensus and feedback from expert groups to accurately reflect clinical characteristics. Furthermore, to develop a practical pattern-identification tool for use in clinical settings, we analyzed the clinical characteristics of 95 patients with FD and assessed the reliability and validity of the newly developed questionnaire to determine how well it could explain and identify the FD subtypes. The entire process of this study is presented in Figure 1.

### 2.1. Development of a Draft of the Standard Tool of Pattern Identification for FD (STPI-FD)

#### 2.1.1. Selection of the Relevant Literature

To develop a draft questionnaire for FD, we collected information on pattern identification types and their associated symptoms through a literature review. We selected studies that presented specific patterns and symptoms of FD, primarily focusing on those published in Korea and China. This included the literature on the frequency of pattern types, clinical guidelines, and clinical trial papers on patients with FD. Studies that did not provide a description of specific pattern identification types and detailed symptoms were excluded.

**Figure 2:**
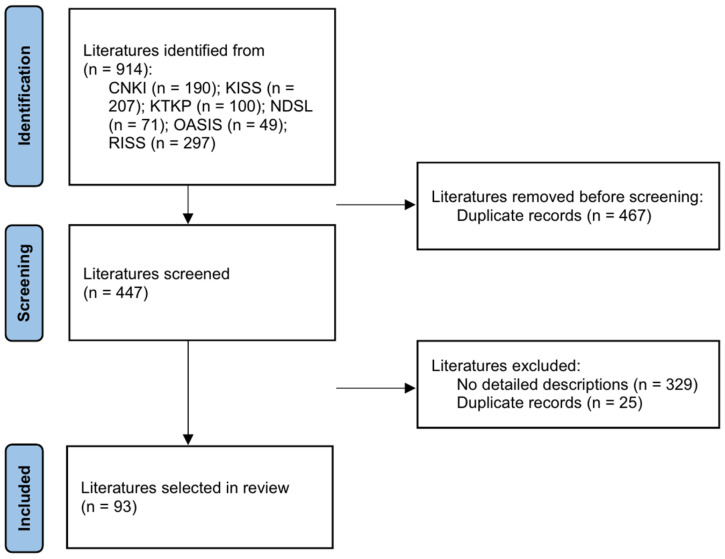
PRISMA flow chart. Abbreviations: CNKI, China National Knowledge Infrastructure Database; KISS, Korean Studies Information Service System; KTKP, Korean Traditional Knowledge Portal; NDSL, National Digital Science Library; OASIS, Oriental Medicine Advanced Searching Integrated System; RISS, Research Information Sharing Service.

Chinese papers published between January 2005 and September 2018 were searched from the China Knowledge Resource Integrated Database using keywords, such as “dyspepsia” and “pattern identification”. After removing duplicate and irrelevant documents, 80 papers were selected from 190 originally identified papers. Korean papers were searched using databases, including the Korean Studies Information Service System, Korean Traditional Knowledge Portal, National Discovery for Science Library, Oriental Medicine Advanced Searching Integrated System, and Research Information Sharing Service, with keywords, such as “indigestion”, “epigastric pain”, and “syndrome differentiation”. Subsequently, a manual search was performed. After excluding duplicates and inappropriate documents, 13 of 724 articles were selected (Figure 2) [22]. Supplement S1 lists the 93 studies assessed in this study.

#### 2.1.2. Selection of Major Pattern Identifications

The frequency of each FD pattern described in the selected literature was analyzed and sorted in order of frequency. Pattern identifications with similar expressions were further integrated through clinical discussions, and those with a frequency of <10% were excluded.

#### 2.1.3. Selection of Major Symptoms

The symptoms associated with pattern identification described in the included studies were categorized as abdominal symptoms, food-related symptoms, mental state, and bowel movements. The frequency of each individual symptom was analyzed and sorted in the order of frequency. Symptoms with a frequency of <10% were excluded.

#### 2.1.4. Korean Translation of Symptoms

A Chinese and classical literature expert specializing in classical Korean medicine and currently serving as a full-time university professor reviewed the translation process for major symptoms. Subsequently, the validity and appropriateness of the Korean version for selected major symptoms were examined.

#### 2.1.5. Evaluation on Importance via Delphi Method

To develop a pattern-identification tool for FD, expert consultation was sought from a panel of professors from the Department of Digestive Diseases at the College of Korean Medicine nationwide, and from Internal Korean Medicine specialists specializing in digestive diseases. Of the 20 experts invited, 14 agreed to participate (response rate: 70.0%). Four expert consultation meetings were conducted (three online, one offline). The Delphi method was applied to evaluate the importance of pattern identification and symptoms, and to confirm the validity of the translations. In cases of disagreement, feedback was provided with the average and individual scores for each item, allowing experts to reassess their ratings and reach a consensus.

(1) Evaluation and Confirmation of Pattern Identification Importance

To determine the inclusion of the FD pattern identification, an initial evaluation of importance was conducted using a 5-point scale on 13 major patterns selected based on their high frequencies. Following the advice of a previous study [23], items with an average score of <3.00, where responses of “not important at all” and “slightly important” were predominant, were excluded owing to low importance. The six final patterns selected through this process are listed in Table 1.

(2) Evaluation and Confirmation of Symptom Importance

The importance of each major symptom within the selected pattern was assessed using a 5-point scale to evaluate its appropriateness. Symptoms with an average score of <3.00 were deemed unsuitable to represent the FD pattern identifications and were therefore excluded. For tongue and pulse diagnosis, the item with the highest score for each type was selected. The average scores and standard deviations of each symptom item were calculated, and their importance values were used to derive item weights based on a formula from a previous study [23]. The final symptoms and weights for pattern identification were subsequently confirmed during expert panel meetings.

(3) Translation Validity Evaluation

The validity of the translations for each symptom was assessed by collecting open-ended feedback during expert panel meetings on the appropriateness and specificity of the translations. The sentences were further revised to be clear and easily understandable to patients, ensuring no ambiguity, after which final modifications were made. Additional feedback included suggestions for using images to depict abdominal locations and stool characteristics to minimize differences in interpretation between patients and evaluators.

#### 2.1.6. Draft of the STPI-FD

A draft of the STPI-FD was completed using the aforementioned process. This draft comprised 38 items selected based on the final six pattern identifications and the most frequently occurring major symptoms within each category. The STPI-FD draft consisted of the following pattern identifications with their respective numbers of items: SSDC, 10 items; spleen deficiency with qi stagnation (SDQS), 10 items; LSDH, 13 items; TACH, 12 items; DHSS, 12 items; and FRDO, 9 items. In accordance with the feedback collected during the expert panel meetings, the symptom evaluation period was set to “the past 2 weeks”. For each of the 38 symptom items, the patients read the questions themselves and responded based on a 5-point scale (0 = never, 1 = almost never, 2 = sometimes, 3 = often, and 4 = always). Tongue and pulse diagnoses were further assessed by a Korean medical doctor who selected the most appropriate response following the examination. The score for each pattern identification was subsequently calculated by multiplying the evaluation score by its respective weight and then summing the total scores of all items within the pattern. The pattern with the highest score was identified as the patient pattern (Table 2).

### 2.2. Evaluation of Clinical Applicability of the Draft of STPI-FD in Clinical Setting

#### 2.2.1. Recruitment of Participants

This study was conducted at two university hospitals, Kyung Hee University Korean Medicine Hospital and Kyung Hee University Hospital at Gangdong, Seoul, Republic of Korea, from 1 May 2019 to 26 May 2020. A total of 95 patients with FD who met the inclusion criteria but not the exclusion criteria were selected as study participants. The inclusion and exclusion criteria were as follows:

(1) Inclusion Criteria
  a. Patients aged 19–75 years diagnosed with FD according to the Rome IV criteria.
  b. Patients with an overall dyspeptic symptom severity of ≥40 assessed on a visual analog scale (VAS, 0–100).
(2) Exclusion Criteria
  a. Organic gastrointestinal causes or alarm symptoms.
  b. Severe structural or psychiatric disorders.
  c. History of gastrointestinal surgery owing to a prior gastrointestinal disease.
  d. Patients who had recently taken medications affecting the gastrointestinal tract, including prokinetic agents, proton pump inhibitors, anti-ulcer drugs, antiemetics, and anticholinergics.

The researchers provided all the participants with a detailed explanation of the study’s purpose and methods, allowing them sufficient time to answer the questions. Participation was voluntary and all participants signed an informed consent form prior to enrollment in the study. This study adhered to the ethical principles of the Declaration of Helsinki and was approved by the institutional review boards of the participating institutions.

#### 2.2.2. Demographics

The demographic characteristics of all the 95 study participants were surveyed using a questionnaire to collect data on sex, age (years), height (cm), weight (kg), and body mass index (BMI) (kg/m^2^). The participants were classified into the following subtypes based on the Rome IV criteria: PDS, EPS, and overlapping types.

#### 2.2.3. Pattern Identification Diagnosis Using STPI-FD

Based on the established inclusion criteria, 95 participants with FD registered in this study completed a draft of the STPI-FD. For the two items requiring a diagnosis by a Korean medicine doctor, each institution employed an independent and trained Korean medicine doctor with at least three years of clinical experience to assess the participants, ensuring objectivity and consistency in the diagnoses. The remaining questionnaire items pertaining to symptoms experienced over the past 2 weeks were self-administered by the participants, who read each question and recorded their responses accordingly. The reliability and validity of the STPI-FD were evaluated based on the data obtained from the participants.

#### 2.2.4. Dyspepsia-Related Scales

We examined the correlations between the STPI-FD and VAS, total dyspepsia symptoms (TDS), single dyspepsia symptoms (SDS), and FD-related quality of life (FD-QoL) scores.

(1) VAS for Dyspepsia

The participants used a continuous line, 100 mm in length, to indicate their subjective overall discomfort from dyspepsia, with “0” representing “no dyspepsia” and “100” indicating “very severe dyspepsia”.

(2) Total Dyspepsia Symptom Scale

The TDS evaluates the severity of dyspeptic symptoms in patients with FD [24]. Symptoms such as postprandial fullness and bloating, early satiation, epigastric pain, epigastric burning, belching, nausea, vomiting, and other dyspepsia-related symptoms over the past two weeks were assessed using a 4-point scale (0 = none, 1 = mild, 2 = moderate, and 3 = severe), with a total score of 24 points.

(3) Single Dyspepsia Symptom Scale

The SDS evaluates the major symptoms of FD (epigastric pain and burning, postprandial fullness, and early satiation) in terms of their frequency, intensity, and discomfort level over the prior 2 weeks using a 4-point scale (0 = never/none, 1 = occasionally/mild, 2 = often/moderate, and 3 = almost daily/severe) [24].

(4) Functional Dyspepsia-Related Quality of Life

Improving the quality of life for patients with FD is often a key objective of treatment. The FD-QoL questionnaire, validated for reliability and validity in Korean patients with FD, assesses the overall quality of life related to FD over the past 2 weeks across four domains: eating, vitality, emotions, and social functioning [25]. It includes twenty-one items divided into five items for eating, four items for vitality, six items for emotions, and six items for social functioning. Each item is rated on a 5-point scale (0 = not at all, 1 = a little, 2 = moderately, 3 = quite a lot, and 4 = very much).

### 2.3. Statistical Analyses

Data are presented as the mean ± standard deviation (SD) or a number (%). To calculate the internal validity of the STPI-FD, the Cronbach’s alpha (α) for all items was determined through reliability analysis. Subsequently, item-total correlation and Cronbach’s alpha were calculated after excluding each item to determine its inclusion based on clinical significance. The Pearson’s chi-square test or Fisher’s exact test were used to analyze the correlation between the pattern-identification items of the FD and Rome IV subtypes, depending on the expected frequency in the cross-tabulation analysis. To analyze the differences in the mean scores of each symptom between the pattern types, parametric tests (one-way analysis of variance) or nonparametric tests (Kruskal–Wallis H test) were conducted based on the normality of the data distribution. Pearson’s correlation analysis was used to examine the correlation between the scores of the pattern-identification items on the FD and other dyspepsia-related scales (VAS, TDS, SDS, and FD-QoL). The Bonferroni correction was applied for multiple testing.

All data organization and statistical analyses were performed using IBM SPSS Statistics for Windows (version 25.0; IBM Corp., Armonk, NY, USA), and statistical significance was set at *p* < 0.05.

## 3. Results

### 3.1. Demographic Characteristics of Patients with FD

A total of 95 participants (25 male and 70 female) were enrolled in this study, and the proportion of women was 2.8 times higher than that of men. The average age of the participants was 47.47 ± 13.57 years, with the highest proportion in their 40s (40–49 years), accounting for 34.74%. The mean BMI was 22.19 ± 3.21 kg/m^2^. Based on the symptoms reported by the participants, the FD subtypes, according to the Rome IV criteria, were as follows: PDS, 36.84%; overlapping, 32.63%; and EPS, 30.53% (Table 3).

### 3.2. Reliability of the STPI-FD

#### 3.2.1. Evaluator-Assessed Items of the STPI-FD

A total of 95 participants evaluated a draft of the STPI-FD. The concordance rates between the patterns identified by the STPI-FD and those identified through tongue and pulse diagnoses by a TKM practitioner were as follows: one pattern (either tongue or pulse diagnosis) matched 31.58% of the cases, while both patterns (tongue and pulse diagnoses) matched 10.53% of the cases. Furthermore, when tongue and pulse diagnostic items were removed from the STPI-FD draft and weights were recalculated, changes in the final identification patterns of the patients were observed in 28.42% of the cases. Thus, the research team excluded the tongue and pulse diagnostic items from the questionnaire to enhance the clinical usability of the STPI-FD by focusing on direct evaluation by the patient and increasing the reliability of the questionnaire.

#### 3.2.2. Patient-Assessed Items of the STPI-FD

The internal consistency of the 38 symptom items in the STPI-FD was evaluated using Cronbach’s alpha (Table 4). Based on the responses of the 95 participants, the overall Cronbach’s alpha for the STPI-FD was 0.941, indicating excellent reliability. Furthermore, each of the six individual pattern identifications showed a Cronbach’s alpha >0.7, ensuring acceptable internal consistency among the items within each pattern category [26].

We reviewed the items with an item-total correlation of ≤0.3, or items whose exclusion increased the Cronbach’s alpha. Q33, “My body feels hot”, has an item-total correlation coefficient of 0.241. Additionally, excluding Q32 “Although I drink cold water because of tightness in my upper abdomen, I dislike cold things and my limbs feel cold” caused no change in the overall Cronbach’s alpha, but slightly increased the Cronbach’s alpha for the TACH from 0.816 to 0.817. Furthermore, based on expert consensus, Q32 ranked seventh among the ten TACH items. After excluding Q32 and Q33, the reliability analysis was repeated. The overall Cronbach’s alpha for the revised STPI-FD increased to 0.942, and the Cronbach’s alpha for half of the six pattern identifications (SDQS, LSDH, TACH) remained above 0.8, confirming that good internal consistency was partially maintained (Table 5). Based on these results, we removed these two items from the questionnaire and recalculated the weight values for each symptom. Consequently, the final version of the STPI-FD consisted of 36 symptom items. This revised tool was used for further validation and statistical analysis to verify clinically significant differences among the pattern identifications. The final STPI-FD and pattern-identification formulae are provided in Supplements S2 and S3, respectively.

### 3.3. Clinical Validity of the STPI-FD

#### 3.3.1. Correlation Analysis Between FD Pattern Identification and Rome IV Subtypes

The overall frequency distributions of pattern identification were as follows: SSDC, 51.6%; DHSS, 14.7%; TACH, 13.7%; SDQS, 9.5%; LSDH, 7.4%; and FRDO, 3.2%. Cross-analysis revealed no significant association between STPI-FD pattern identification and Rome IV subtype (*p* = 0.289). However, specific subtypes demonstrated higher frequencies within each identified pattern: PDS was predominant in SDQS (55.6%) and FRDO (100.0%); EPS was predominant in LSDH (57.1%); and the overlapping subtype was most frequently observed in DHSS (50.0%) (Table 6).

#### 3.3.2. Analysis of Symptom Scores Across Pattern Identifications

An analysis was conducted to determine whether there were any significant differences in the scores for each symptom item across different pattern identifications (Table 7). The results indicated significant differences in the mean scores of the following items among the pattern identifications: Q7, Q8, Q11, Q17, Q20, Q21, Q22, Q26, Q27, Q30, Q37, and Q38.

The results of the Bonferroni correction method for significant mean score differences were as follows: the mean rank for Q21 was significantly higher in the SDQS (77.56) compared with the TACH (39.38) (*p* < 0.05) and the SSDC (39.86) (*p* < 0.01); the mean rank for Q27 was significantly higher in the SDQS (73.44) compared with the LSDH (20.29) (*p* < 0.01); the mean rank for Q30 was significantly higher in the SSDC (58.40) compared with the LSDH (22.36) (*p* < 0.05) and the TACH (23.58) (*p* < 0.001); and the mean rank for Q37 was significantly higher in the DHSS (67.79) compared with the LSDH (29.57) (*p* < 0.05) and the TACH (32.19) (*p* < 0.01).

#### 3.3.3. Correlation Analysis of STPI-FD and Other Dyspepsia-Related Questionnaires

**Figure 3:**
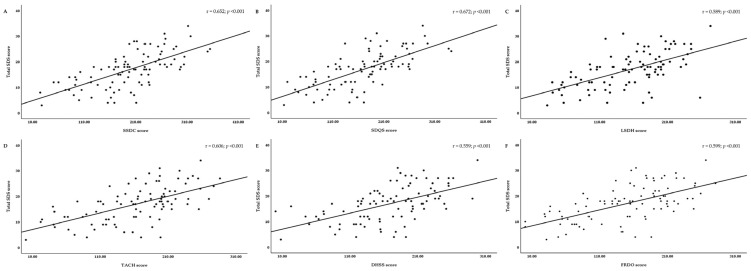
Scatter plots of the total SDS score against each pattern-identification score. (**A**) SSDC; (**B**) SDQS; (**C**) LSDH; (**D**) TACH; (**E**) DHSS; (**F**) FRDO. Abbreviations. DHSS, dampness and heat in the spleen and stomach; FRDO, food retention disorder; LSDH, liver–stomach disharmony; SDQS, spleen deficiency with qi stagnation; SDS, single dyspepsia symptoms; SSDC, spleen and stomach deficiency and cold; TACH, tangled cold and heat. Correlation coefficients (r) were analyzed using Pearson’s correlation analysis. Values between 0.6 and 0.8 indicate a strong correlation (**A**,**B**,**D**), while values between 0.4 and 0.6 indicate a moderate correlation (**C**,**E**,**F**). Results were considered statistically significant at *p* < 0.000125.

The correlation between the scores of each pattern-identification type calculated using the STPI-FD and the scores of the existing dyspepsia-related scales (VAS, TDS, SDS, and FD-QoL) was investigated based on the responses of the 95 study participants to evaluate concurrent validity (Table 8). Correlation analysis revealed significant positive correlations among most of the indices, except between the epigastric burning score and the SSDC and SDQS scores. The correlation coefficients (r) between the SDQS and total SDS scores, SSDC and total SDS scores, and TACH and total SDS scores were 0.672 (*p* < 0.000125), 0.652 (*p* < 0.000125), and 0.606 (*p* < 0.000125), respectively, indicating a strong relationship (Figure 3).

## 4. Discussion

### 4.1. Summary of the Study

In this study, we developed a Korean version of the STPI-FD, a questionnaire comprising six patterns and 36 items. Each item in the questionnaire demonstrated high overall reliability (Cronbach’s alpha = 0.942); however, Cronbach’s alpha exceeded 0.8 in only three of the six pattern types. The validity of the STPI-FD was confirmed through the distribution of FD subtypes across pattern identifications, comparisons of item scores among pattern identifications, and correlation analyses between pattern identification scores and existing dyspepsia-related questionnaires. These findings indicate that STPI-FD is partially useful and clinically beneficial for classifying and diagnosing FD subtypes in patients treated with TKM.

### 4.2. Clinical Application of the Study

Pattern identification is distinct from clinical symptoms, and it can be combined with modern clinical medicine’s biomedical diagnosis. Information collected for pattern identification typically includes subjective symptoms and signs, such as findings from abdominal examinations, tongue appearance, and pulse strength. Pattern identification can be used to categorize a patient’s condition and specific disease stage, allowing clinicians to select herbal medicine or acupuncture treatment based on established criteria. It can also be performed to observe and record patient responses and treatment efficacy [11]. Even for the same disease, different pattern types may require different treatments and responses, and similar prescriptions may be administered if the pattern type is the same, even across different disease groups [27]. Therefore, consistent evaluation indicators are essential in clinical TKM for treatment decisions through pattern identification. These indicators help minimize inter-evaluator variability, overcome subjectivity, and provide evidence supporting reliability through standardization and reproducibility enhancement through education [28]. Recent efforts in Korea have focused on developing standardized tools and questionnaires for pattern identification. Research has been conducted to develop and establish foundational studies for pattern-identification tools based on the characteristics of various diseases, such as gastroesophageal reflux disease [23], common cold [29], polycystic ovary syndrome [30], and depression [31]. These diagnostic tools have undergone reliability and validity assessments among patients [32,33,34], and studies have analyzed their correlation with objective measurement indicators to enhance their clinical applicability [35,36]. The development of disease-specific pattern identification tools through standardized methods can aid not only clinical settings but also help in designing clinical trials with better and higher efficacy strategies.

Research on pattern identification in FD has highlighted that the efficacy of TKM treatments may vary based on subtypes, even among patients diagnosed with FD according to the same Rome criteria. In a study investigating the efficacy of Naesohwajung-tang administered to 116 patients with FD over 4 weeks, significant improvements were observed compared to a placebo. Furthermore, subgroup analysis revealed that patients diagnosed with the food retention type and those classified as DHSS showed particularly notable symptom improvements compared to placebo [37]. Treatment can also be differentiated based on pattern identification. In one study involving 70 patients with FD, acupoints were selected based on syndrome differentiation (deficiency or excess syndrome), and patients received treatment for 20 days, achieving improvements in both short- and long-term symptoms [38]. Additionally, applying pattern identification at diagnosis, 96 patients with FD with liver stagnation and spleen deficiency were treated with the “experienced ten acupoints” five times a week for 2 weeks. This treatment achieved greater symptom score reductions and higher total effective rates compared to the irrelevant acupoint prescription control group [39]. Similarly, in a study of 160 patients with FD classified as SDQS, administration of the modified LiuJunZi decoction for 4 weeks resulted in significant symptom improvements and enhanced gastric emptying compared to placebo [24]. These studies demonstrate that applying subtype differentiation in a clinical setting for patients with FD can lead to improved therapeutic outcomes and patient compliance, as well as offering a wider range of treatment options for healthcare providers, with potential financial and ethical benefits on a socioeconomic level.

Despite the clinical value of pattern identification in FD, variations exist in the names, numbers, and criteria of pattern identification, as well as in the symptom items included across different studies. This highlights the need for the development and evaluation of reliable and valid pattern-identification tools for TKM. Therefore, we aimed to develop a standard questionnaire to subgroup patients with FD by extracting pattern identification indicators from the historical literature, recent clinical trial papers, and official clinical practice guidelines in traditional medicine. This approach was designed to comprehensively cover the complex and diverse conditions of the disease and to establish clinical evidence and diagnostic bases for pattern identification. Open-ended responses were collected from an expert advisory committee, using the Delphi method. Finally, by applying the developed pattern-identification tool to patients with FD, we sought to identify the clinical characteristics associated with each pattern and symptom. This study provided a basis for the practical use of this tool as an objective diagnostic and treatment evaluation instrument in clinical practice.

In the present study, we conducted a systematic literature review, primarily focusing on clinical papers from domestic and international databases, to identify the major pattern items and symptoms related to FD. We referenced 93 papers published in Korea and China over the last decade, mostly from 2005 onward, as identified in a previous study [21]. Among them, 13 were from Korea and 80 were from China. Subsequently, the frequencies of the extracted patterns and symptoms were reviewed, followed by an evaluation of their importance by an expert panel using the Delphi method. This process aimed to finalize a draft of the pattern-identification tool, including item weights and calculation formulas, based on the symptoms observed in clinical practice. To enhance the utility of these tools in clinical settings, emphasis has been placed on differentiating diagnostic types, and feedback from expert advisory meetings has been actively incorporated. For example, symptom Q8 in the SDQS had an average importance score of 2.82, which did not meet the selection criterion of 3.00. Nonetheless, it was ultimately included in the pattern identification items because it was recognized during advisory meetings as an important symptom distinguishing it from the SSDC.

The draft STPI-FD, developed using the Delphi method, consisted of 38 items. All symptoms were self-reported by the participants, with two additional items evaluated by practitioners using a 5-point Likert scale. This structure was inspired by previous studies that used weights to achieve uniformity in questionnaire composition, scoring, and ease of completion [23]. During consultation with the advisory committee, the importance of recognizing acute symptoms, even within the chronic context of FD, was emphasized. Consequently, the symptom assessment period was limited to the previous 2 weeks, aligning with the typical 2–4-week survey periods used in other dyspepsia-related scales [24,25]. The final score was calculated by multiplying each item score by its weight, with the highest total score indicating the primary pattern of the patient.

To preliminarily assess the clinical significance and diagnostic appropriateness of the developed pattern-identification tool, 95 adult patients diagnosed with FD according to the Rome criteria were enrolled. During draft development of the STPI-FD, items related to tongue and pulse diagnoses were included. However, these items compromised the reliability when applied to actual patients and were subsequently excluded from the final tool. Tongue and pulse diagnoses are distinctive aspects of TKM; however, their subjective nature results in a mismatch with the final pattern identification type in approximately 70% of cases, likely due to nonstandardized evaluation methods and varying diagnostic skills among clinicians. To reduce evaluator bias, standardized education, consensus among clinicians, and the use of reliable automatic diagnostic devices, particularly for tongue and pulse analysis, should be considered. In this study, by excluding tongue and pulse diagnostic items, the STPI-FD was simplified, allowing patients to complete the tool within <5 min without requiring an evaluator. This modification maintained the reliability of the tool, while enhancing its practicality and ease of use in clinical settings.

Reliability refers to the consistency of a test in measuring the same concept across items. Internal consistency reliability was assessed using Cronbach’s alpha [40], where a value of ≥0.7 is considered acceptable, and ≥0.8 indicates a good tool [41]. In this study, the initial 38-item version of the tool demonstrated a high Cronbach’s alpha of 0.941, with acceptable coefficients for each pattern type. During the development process, items Q33 and Q32 were removed to enhance reliability. Q33, included only in the DHSS, had an item-total correlation of 0.241, which was below the threshold of 0.3, indicating low consistency. It was also ranked last in importance within the DHSS based on expert consensus and had a low patient evaluation score, leading to its exclusion. Removing Q33 slightly increased the overall Cronbach’s alpha to 0.942 and the DHSS alpha from 0.750 to 0.759. Similarly, Q32, part of the TACH, was removed, as its exclusion slightly increased the TACH alpha from 0.799 to 0.817, with minimal impact on the overall reliability. After excluding these items, the final 36-item tool had an overall Cronbach’s alpha of 0.942, confirming a strong internal consistency. The Cronbach’s alpha for each pattern type ranged from 0.737 to 0.841, indicating acceptable or good internal consistency.

Validity refers to the degree to which a test accurately measures its intended variables and can be categorized into content, criterion-related, and construct validity [42]. In this study, we evaluated the clinical validity of the pattern-identification tool by analyzing the clinical aspects of pattern identification, examining the characteristics of the constituent items, and comparing item scores across different patterns. Clinically, according to the final version of STPI-FD classification, the SSDC type accounted for slightly more than half (51.6%) of the 95 patients with FD, while other types accounted for around 10% each. FD is a chronic disease with symptoms that vary over time and can lead to fatigue, decreased appetite, and weight loss due to its chronic nature [1]. The SSDC type includes symptoms such as eating small amounts, mental exhaustion, and weakness, which are closely related to the prognosis and course of FD, explaining why it is prevalent among FD patients. Additionally, we analyzed the clinical correlation between the pattern types and Rome IV subtypes. No statistically significant correlations were found. However, PDS, EPS, and overlapping types were most frequently observed in SDQS, FRDO, LSDH, and DHSS, suggesting a potential link to Rome IV subtypes. For instance, key symptoms, such as Q19 “feeling more tightness after eating” in SDQS and Q18 “loss of appetite” in FRDO, correlated with postprandial fullness and early satiety, aligning with PDS. Similarly, symptoms like Q3 “upper abdominal pain”, Q5 “soreness in the chest and upper abdomen”, and Q6 “acid reflux” in LSDH were associated with epigastric pain and burning, consistent with the pattern observed in EPS. The analysis of individual symptoms within each pattern identified significant differences in 12 items across the six patterns, with 4 items showing particularly distinct results in the post-hoc analysis. These findings indicate the distinct characteristics associated with each pattern, reinforcing the clinical utility of the STPI-FD in differentiating FD subtypes based on TKM diagnostic criteria. Identifying these significant differences underscores the potential for more precise and individualized treatment approaches for FD tailored to specific pattern identifications.

Concurrent validity was further analyzed by examining the correlations between the pattern-identification tool and existing dyspepsia-related scales, including the VAS, TDS, SDS, and FD-QoL. Assessing the state of FD through symptom evaluation is crucial. The TDS is a questionnaire that assesses the severity of eight gastrointestinal symptoms and has demonstrated strong correlations with the Nepean Dyspepsia Index, a validated questionnaire for FD, making it a key tool for assessing symptom changes in FD patients [43]. The SDS is a concise, self-reported measure that evaluates the four major symptoms used in the diagnosis of FD, and it aims to assess the suitability of the STPI-FD as an FD-specific questionnaire. Both the TDS and SDS have been widely utilized in various studies to evaluate the effects of herbal medicine on FD [24,37,44,45]. In this study, the VAS was employed as an inclusion criterion to evaluate the severity of dyspepsia. Pattern identification is regarded as an immediate and dynamic approach, responding sensitively to changes in clinical status, which makes the VAS a well-accepted tool for symptom assessment in FD [43]. The analysis revealed significant positive correlations, with most coefficients indicating a moderate correlation, ranging from 0.4 to 0.6 [46]. Notably, epigastric burning was more strongly correlated with the LSDH and TACH patterns, consistent with the prevalence of EPS in these subtypes. FD is often accompanied by other physical symptoms, such as sleep disorders [47], irritable bowel syndrome [48], and fatigue [49]. The strong correlation with the FD-QoL indicates that the pattern-identification tool effectively reflects overall physical and gastrointestinal symptoms, distinguishing it from other dyspepsia questionnaires. This underscores the importance of including systemic symptoms in pattern identification to ensure a more detailed and holistic assessment of the patient’s condition. In conclusion, this study systematically identified patterns and symptoms related to FD, thereby contributing to the standardization of syndrome differentiation. The developed questionnaire is user-friendly, allowing patients to complete it independently, making it a practical tool in clinical settings. Future adaptations could include objective indicators and simplified language to enhance ease of use and accuracy.

### 4.3. Limitations of the Study

This study had certain limitations. First, a significant limitation is the relative scarcity of Korean research in the literature review, which led to pattern identification and symptoms being primarily derived from Chinese literature. This limits the applicability of our findings to domestic clinical settings. Although domestic expert meetings and the Delphi method were used to mitigate this issue, future updates should incorporate a broader range of studies, including both domestic and multinational studies, to reduce regional bias. Second, essential observational elements of TKM, such as pulse, tongue, and abdominal examinations, which are typically performed by Korean medicine practitioners, were excluded from the study. Future research should consider integrating objective examination indicators such as tongue and pulse diagnostic devices, gastric emptying tests, and electrogastrography into the diagnostic tool. Alternatively, to standardize these diagnostic practices, detailed guidelines should be developed, and expert training should be implemented to ensure consistency and reliability across practitioners. Finally, the study included 95 participants, which may not have been sufficient for a comprehensive assessment of reliability and validity in a clinical setting. Previous research suggests that a sample size of at least 400 cases is necessary for accurate evaluation [50]. Furthermore, internal consistency was not satisfactory in some patterns. To improve internal consistency, it is crucial to minimize the statistical heterogeneity between the symptoms and weights determined by experts and the frequency and severity of symptoms reported by patients. This can be achieved by distinguishing between FD-specific and FD-non-specific symptoms and identifying unique combinations within the questionnaire. Future research should utilize a larger sample size to enable more precise verification of the tool’s reliability and validity. Additionally, comparing the diagnostic consistency of the questionnaire with that of a healthy control group will provide further validation. The use of receiver operating characteristic (ROC) curves is recommended to compare the results of the STPI-FD with pattern identification types diagnosed by multiple physicians without the aid of a questionnaire. This approach will help determine the optimal cutoff points for sensitivity and specificity for each pattern type.

### 4.4. Significance of the Study

This study introduced a diagnostic tool for FD patterns, which includes items that assess specific symptoms of FD and the overall condition of the patient. The tool was developed through a systematic literature review and expert consensus. Preliminary testing in 95 patients with FD partially demonstrated the tool’s reliability, validity, and clinical applicability. However, addressing the limitations identified in this study is crucial for standardizing and objectifying pattern identification in future research. The data generated can serve as foundational evidence for evidence-based medicine, contribute to clinical statistical research, and support the development of machine learning algorithms for medical diagnostics. Furthermore, this study provides evidence to support the clinical efficacy of syndrome differentiation in subgrouping and treating FD, which can inform clinical practice guidelines and optimize the utilization of medical resources for patients with FD.

## 5. Conclusions

This study successfully developed a 36-item diagnostic tool to identify FD patterns and analyze their clinical characteristics. Although some aspects of internal consistency did not reach significant levels, the STPI-FD demonstrated high validity, establishing it as a potential tool for diagnosing FD subtypes in TKM. Its application is expected to enhance diagnostic accuracy, inform treatment decisions, and facilitate the quantitative evaluation of FD prognosis for each pattern. Future research should focus on verifying the cut-off values for each pattern type and confirming their reliability and specificity through larger-scale studies.

## Figures and Tables

**Table 1:** The final selected pattern identification items.

Pattern Identification Item	Average Score (Mean ± SD)	Rank
LSDH	4.21 ± 1.25	1
FRDO	4.00 ± 1.24	2
SSDC	3.86 ± 1.41	3
SDQS	3.50 ± 1.29	4
TACH	3.14 ± 1.03	5
DHSS	3.07 ± 1.21	6

Abbreviations. DHSS, dampness and heat in the spleen and stomach; FRDO, food retention disorder; LSDH, liver–stomach disharmony; SD, standard deviation; SDQS, spleen deficiency with qi stagnation; SSDC, spleen and stomach deficiency and cold; TACH, tangled cold and heat.

**Table 2:** Draft version of the STPI-FD.

No.	Symptoms	Mean ± SD	Weighted Value
**SSDC**			
Q1	My upper abdomen feels tight and occasionally mildly painful.	4.00 ± 1.00	8.27
Q10	I have no appetite, but my mouth is not dry.	3.36 ± 0.81	8.59
Q17	I eat small amounts and feel full easily.	4.09 ± 0.70	12.07
Q25	My complexion is pale and sometimes turns yellow.	4.00 ± 0.89	9.24
Q26	I feel mentally exhausted and my whole body is weak.	4.27 ± 0.90	9.76
Q30	My limbs feel weak, and my hands and feet are cold.	4.45 ± 0.69	13.39
Q31	My pain decreases when my abdomen is warmed or massaged.	3.82 ± 0.87	9.03
Q35	My stools are loose.	3.36 ± 1.03	6.77
T1	Pale tongue with white fur, with teeth marks on the margins.	3.82 ± 0.60	13.09
P1	Fine-weak or slowdown pulse.	3.73 ± 0.79	9.80
**SDQS**			
Q3	My upper abdomen feels bloated and occasionally painful.	4.00 ± 1.00	9.10
Q8	My chest feels tight.	2.82 ± 0.60	10.63
Q19	I have no appetite and feel tightness after eating.	4.09 ± 0.70	13.28
Q21	I belch and hiccup frequently.	3.09 ± 0.70	10.04
Q24	My face lacks luster or color.	3.27 ± 0.65	11.51
Q26	I feel mentally exhausted and my whole body feels weak.	3.55 ± 0.93	8.63
Q28	My chest feels hot and tight, and I get angry easily.	3.18 ± 0.98	7.37
Q35	My stools are loose.	3.09 ± 0.70	10.04
T2	Pale tongue with thin white fur.	3.45 ± 0.69	11.43
P2	Fine and string-like pulse.	2.91 ± 0.83	7.96
**LSDH**			
Q3	My upper abdomen feels bloated and occasionally painful.	3.91 ± 0.83	7.93
Q5	My chest and upper abdomen feel sore or painful, and I feel hungry, making me uncomfortable.	3.55 ± 0.82	7.29
Q6	Acid reflux makes my stomach feel sore.	4.27 ± 0.90	7.97
Q8	My chest feels tight.	3.82 ± 0.60	10.68
Q9	My flank feels bloated or painful.	4.27 ± 0.65	11.15
Q11	My mouth is dry or bitter.	3.36 ± 0.92	6.14
Q13	I sometimes feel nauseous or vomit.	3.00 ± 0.63	8.00
Q18	I do not feel like eating.	3.09 ± 0.70	7.44
Q21	I frequently belch and hiccup.	3.45 ± 0.82	7.11
Q23	I sigh often.	3.18 ± 0.75	7.15
Q28	My chest feels hot and tight, and I get angry easily.	4.18 ± 0.98	7.19
T4	Pale red tongue with thin white fur.	2.82 ± 0.98	4.84
P3	String-like pulse	3.45 ± 0.82	7.11
**TACH**			
Q2	My upper abdomen feels tight, and occasionally severely painful.	3.55 ± 1.21	5.79
Q5	My chest and upper abdomen feel sore or painful or hungry, making me uncomfortable.	3.82 ± 0.87	8.66
Q6	Acid reflux makes my stomach feel sore.	3.09 ± 0.94	6.49
Q7	My stomach feels bloated and gurgles.	3.64 ± 0.67	10.70
Q11	My mouth is dry or tastes bitter.	3.55 ± 0.69	10.23
Q16	Acid frequently regurgitates into my mouth.	3.18 ± 0.87	7.22
Q18	I do not feel like eating.	3.09 ± 0.54	11.36
Q27	I feel heat and tightness in my chest, occasionally accompanied by a feverish sensation throughout my body.	3.82 ± 0.75	10.09
Q32	Although I drink cold water because of tightness in my upper abdomen, I dislike cold things and my limbs feel cold.	3.55 ± 0.93	7.53
Q35	My stools are loose.	3.73 ± 0.90	8.17
T3	Pale tongue with yellow fur.	2.91 ± 0.70	8.23
P3	String-like pulse.	2.91 ± 1.04	5.52
**DHSS**			
Q2	My upper abdomen feels tight and occasionally severely painful.	3.73 ± 0.65	10.72
Q5	My chest and upper abdomen feel sore or painful or hungry, making me uncomfortable.	3.18 ± 0.75	7.89
Q6	Acid reflux makes my stomach feel sore.	3.64 ± 1.03	6.59
Q12	My mouth is dry or bitter, but I do not feel like drinking water.	3.64 ± 0.81	8.36
Q13	I sometimes feel nauseous or vomit.	3.27 ± 1.01	6.03
Q20	I belch frequently.	3.18 ± 0.60	9.82
Q29	My body feels heavy and lethargic.	3.64 ± 1.12	6.04
Q33	My body feels hot.	3.00 ± 0.77	7.21
Q34	My urine is dark and scanty.	3.55 ± 0.69	9.59
Q37	My stools are hard or loose, and I do not feel relieved after a bowel movement.	3.36 ± 0.50	12.40
T5	Red tongue with yellow-slimy fur.	3.91 ± 0.83	8.75
P4	Slippery pulse.	3.64 ± 1.03	6.59
**FRDO**			
Q4	My upper abdomen feels heavy and painful, similar to indigestion, and the pain worsens when my abdomen is pressed.	4.73 ± 0.47	25.19
Q14	I sometimes feel nauseous or vomit, and my symptoms reduce after vomiting.	3.91 ± 0.83	11.71
Q15	I have vomited undigested food.	3.09 ± 1.04	7.37
Q18	I do not feel like eating.	4.09 ± 0.94	10.79
Q22	Belching produces a foul smell and acid regurgitation.	4.00 ± 1.00	9.96
Q36	I have difficulty with bowel movements or experience diarrhea.	3.27 ± 1.01	8.07
Q38	My flatulence smells bad.	3.00 ± 1.00	7.47
T6	Thick slimy fur.	3.73 ± 1.01	9.19
P5	Slippery and replete pulse.	3.50 ± 0.85	10.25

Abbreviations. DHSS, dampness and heat in the spleen and stomach; FRDO, food retention disorder; LSDH, liver–stomach disharmony; SD, standard deviation; SDQS, spleen deficiency with qi stagnation; SSDC, spleen and stomach deficiency and cold; STPI-FD, standard tool for pattern identification for functional dyspepsia; TACH, tangled cold and heat.

**Table 3:** General characteristics of the study participants.

Characteristics	Total (*n* = 95)
Sex (%)	
Male	25 (26.32)
Female	70 (73.68)
Age (years)	47.47 ± 13.57
BMI (kg/m^2^)	22.19 ± 3.21
FD subtypes (%)	
PDS	35 (36.84)
EPS	29 (30.53)
Overlapping	31 (32.63)

Abbreviations. BMI, body mass index; EPS, epigastric pain syndrome; FD, functional dyspepsia; PDS, postprandial distress syndrome.

**Table 4:** Mean scores and internal consistency of the symptoms included in the STPI-FD (*n* = 95).

Items	Mean ± SD	Symptomatic (*n*)	Asymptomatic (*n*)	Item-Total Correlation	Cronbach’s α When the Item Was Deleted
Q1	2.13 ± 0.97	86	9	0.607	0.939
Q2	1.43 ± 0.88	77	18	0.617	0.939
Q3	1.74 ± 0.98	82	13	0.655	0.939
Q4	1.75 ± 1.02	83	12	0.617	0.939
Q5	2.06 ± 0.97	87	8	0.571	0.939
Q6	1.56 ± 0.95	79	16	0.471	0.940
Q7	1.99 ± 1.07	84	11	0.671	0.939
Q8	1.92 ± 0.98	85	10	0.614	0.939
Q9	1.14 ± 0.87	71	24	0.562	0.940
Q10	1.46 ± 0.98	81	14	0.462	0.940
Q11	1.52 ± 0.98	76	19	0.459	0.940
Q12	1.20 ± 1.04	66	29	0.354	0.941
Q13	1.13 ± 0.93	65	30	0.516	0.940
Q14	1.17 ± 1.09	61	34	0.429	0.941
Q15	1.21 ± 0.93	71	24	0.498	0.940
Q16	1.41 ± 0.96	77	18	0.602	0.939
Q17	2.28 ± 1.06	93	2	0.461	0.940
Q18	1.23 ± 1.03	68	27	0.476	0.940
Q19	1.52 ± 1.13	76	19	0.616	0.939
Q20	2.02 ± 1.26	83	12	0.544	0.940
Q21	1.33 ± 0.94	75	20	0.508	0.940
Q22	1.38 ± 1.10	68	27	0.651	0.939
Q23	1.87 ± 1.15	81	14	0.491	0.940
Q24	1.73 ± 1.22	80	15	0.562	0.940
Q25	1.37 ± 1.18	70	25	0.639	0.939
Q26	2.27 ± 1.09	91	4	0.638	0.939
Q27	1.69 ± 1.14	80	15	0.560	0.940
Q28	1.68 ± 1.07	81	14	0.678	0.939
Q29	2.42 ± 1.02	92	3	0.599	0.939
Q30	2.26 ± 1.21	87	8	0.498	0.940
Q31	2.13 ± 1.01	88	7	0.428	0.941
Q32	1.71 ± 1.19	79	16	0.438	0.941
Q33	1.33 ± 1.12	68	27	0.241	0.942
Q34	1.61 ± 1.02	80	15	0.374	0.941
Q35	1.52 ± 1.13	76	19	0.461	0.940
Q36	1.46 ± 1.07	77	18	0.490	0.940
Q37	1.65 ± 1.20	77	18	0.562	0.940
Q38	1.75 ± 1.16	81	14	0.570	0.939

Abbreviations. SD, standard deviation; STPI-FD, standard tool of pattern identification for functional dyspepsia.

**Table 5:** Revised internal consistency in each pattern identification item, excluding Q32 and Q33 (*n* = 95).

Pattern Identification	Number of Items	Cronbach’s α
SSDC	8	0.794
SDQS	8	0.811
LSDH	11	0.841
TACH	9	0.817
DHSS	9	0.759
FRDO	7	0.737

Abbreviations. DHSS, dampness and heat in the spleen and stomach; FRDO, food retention disorder; LSDH, liver–stomach disharmony; SDQS, spleen deficiency with qi stagnation; SSDC, spleen and stomach deficiency and cold; TACH, tangled cold and heat.

**Table 6:** Distribution of FD subtypes within each pattern identification (*n* = 95).

Pattern Identification	Frequency (%)	FD Subtypes			Total	Percentage (%)	Fisher’s Exact Test (*p*)
		PDS	EPS	Overlapping			
SSDC	Frequency % Within	19 (38.8)	13 (26.5)	17 (34.7)	49 (100.0)	51.6	0.289
SDQS	Frequency % Within	5 (55.6)	3 (33.3)	1 (11.1)	9 (100.0)	9.5	
LSDH	Frequency % Within	2 (28.6)	4 (57.1)	1 (14.3)	7 (100.0)	7.4	
TACH	Frequency % Within	3 (23.1)	5 (38.5)	5 (38.5)	13 (100.0)	13.7	
DHSS	Frequency % Within	3 (21.4)	4 (28.6)	7 (50.0)	14 (100.0)	14.7	
FRDO	Frequency % Within	3 (100.0)	0 (0.0)	0 (0.0)	3 (100.0)	3.2	
Total	Frequency % Within	35 (36.8)	29 (30.5)	31 (32.6)	95 (100.0)	100.0	

Abbreviations. DHSS, dampness and heat in the spleen and stomach; EPS, epigastric pain syndrome; FD, functional dyspepsia; FRDO, food retention disorder; LSDH, liver–stomach disharmony; PDS, postprandial distress syndrome; SDQS, spleen deficiency with qi stagnation; SSDC, spleen and stomach deficiency and cold; TACH, tangled cold and heat.

**Table 7:** Comparison of symptom scores among different pattern identifications (*n* = 95).

Items	Mean Rank						χ^2^	*p*	Bonferroni
	SSDC (*n* = 49)	SDQS (*n* = 9)	LSDH (*n* = 7)	TACH (*n* = 13)	DHSS (*n* = 14)	FRDO (*n* = 3)			
Q1	51.06	56.44	31.43	37.08	52.00	40.00	7.683	0.175	NA
Q2	47.40	53.33	44.86	42.19	52.93	51.33	1.750	0.883	NA
Q3	49.13	57.67	34.14	38.77	49.79	64.50	6.147	0.292	NA
Q4	47.39	54.33	35.43	44.96	49.75	73.33	5.093	0.405	NA
Q5	44.79	55.17	52.93	49.04	52.18	43.50	2.211	0.819	NA
Q6	42.17	49.78	48.21	58.77	56.64	50.33	6.204	0.287	NA
**Q7**	**43.19**	**63.89**	**32.43**	**47.54**	**58.86**	**66.50**	**11.174**	0.048 *	**NA**
**Q8**	**44.16**	**68.22**	**33.79**	**44.88**	**60.46**	**38.50**	**12.508**	0.028 *	**NA**
Q9	41.99	62.39	44.64	47.12	60.71	55.33	9.079	0.106	NA
Q10	49.19	51.33	35.14	41.54	49.93	67.50	4.482	0.482	NA
**Q11**	**42.00**	**61.33**	**36.43**	**65.04**	**47.50**	**61.50**	**12.585**	0.028 *	**NA**
Q12	45.49	52.89	36.00	49.77	58.86	44.00	4.672	0.457	NA
Q13	43.43	54.61	51.71	39.46	58.82	80.67	10.621	0.059	NA
Q14	42.56	63.61	47.86	42.58	55.82	77.33	10.711	0.057	NA
Q15	42.28	60.28	45.29	48.77	52.82	85.17	10.853	0.054	NA
Q16	42.96	59.39	40.00	51.62	54.68	68.00	7.063	0.216	NA
**Q17**	**56.58**	**49.39**	**29.29**	**32.92**	**41.89**	**41.17**	**13.752**	0.017 *	**NA**
Q18	50.35	54.61	37.36	38.73	50.54	43.00	3.911	0.562	NA
Q19	46.66	65.89	39.07	41.73	52.14	44.83	6.094	0.297	NA
**Q20**	**44.17**	**61.89**	**35.86**	**36.35**	**64.82**	**69.17**	**14.596**	0.012 *	**NA**
**Q21**	**39.86**	**77.56**	**47.79**	**39.38**	**59.46**	**76.67**	**23.666**	0.000 ***	a < b ** d < b *
**Q22**	**41.33**	**68.89**	**42.86**	**44.38**	**58.64**	**72.33**	**13.942**	0.016 *	**NA**
Q23	48.39	60.72	50.79	30.46	56.54	33.17	10.112	0.072	NA
Q24	52.47	64.78	34.14	34.54	42.71	40.00	10.933	0.053	NA
Q25	53.77	59.00	33.50	34.77	40.64	46.33	10.265	0.068	NA
**Q26**	**53.68**	**57.28**	**27.79**	**73.44**	**45.14**	**50.17**	**11.380**	0.044 *	**NA**
**Q27**	**47.12**	**73.44**	**20.29**	**53.46**	**43.32**	**48.83**	**16.804**	0.005 **	c < b **
Q28	43.95	65.78	42.86	44.54	52.61	66.33	7.522	0.185	NA
Q29	49.96	54.22	28.29	36.77	55.86	55.33	8.515	0.130	NA
**Q30**	**58.40**	**48.83**	**22.36**	**23.58**	**44.93**	**55.67**	**24.966**	0.000 ***	c < a * d < a ***
Q31	51.83	43.06	34.43	44.46	46.68	53.50	3.689	0.595	NA
Q34	47.36	53.22	43.64	36.27	61.04	43.00	6.671	0.246	NA
Q35	47.37	68.50	39.07	48.65	38.07	61.17	8.865	0.115	NA
Q36	47.57	54.39	36.86	41.38	50.54	78.67	6.754	0.240	NA
**Q37**	**45.74**	**58.11**	**29.57**	**32.19**	**67.79**	**73.67**	**19.970**	0.001 **	c < e * d < e **
**Q38**	**44.55**	**63.28**	**34.64**	**39.65**	**63.21**	**54.83**	**11.510**	0.042 *	**NA**

Abbreviations. DHSS, dampness and heat in the spleen and stomach; FRDO, food retention disorder; LSDH, liver–stomach disharmony; NA, not applicable; SDQS, spleen deficiency with qi stagnation; SSDC, spleen and stomach deficiency and cold; TACH, tangled cold and heat. (a) SSDC; (b) SDQS; (c) LSDH; (d) TACH; (e) DHSS. Statistically significant values, highlighted in bold, were determined using the Kruskal–Wallis test (* *p* < 0.05, ** *p* < 0.01, and *** *p* < 0.001).

**Table 8:** Correlation coefficients between the six pattern identifications of the STPI-FD and other dyspepsia-related questionnaires (*n* = 95).

Items	TDS	SDS					VAS	FD-QoL
		Total	Epigastric Pain	Epigastric Burning	Postprandial Fullness	Early Satiety		
**SSDC**	0.518 ***	0.652 ***	0.544 ***	0.250	0.539 ***	0.506 ***	0.537 ***	0.559 ***
SDQS	0.587 ***	0.672 ***	0.504 ***	0.256	0.591 ***	0.546 ***	0.516 ***	0.580 ***
LSDH	0.521 ***	0.589 ***	0.441 ***	0.454 ***	0.418 ***	0.383 **	0.337 **	0.405 ***
TACH	0.561 ***	0.606 ***	0.471 ***	0.439 ***	0.442 ***	0.391 ***	0.385 ***	0.426 ***
DHSS	0.551 ***	0.559 ***	0.427 ***	0.328 **	0.435 ***	0.404 ***	0.371 **	0.456 ***
FRDO	0.544 ***	0.599 ***	0.451 ***	0.391 ***	0.474 ***	0.403 ***	0.452 ***	0.462 ***

Abbreviations. DHSS, dampness and heat in the spleen and stomach; FD-QoL, functional dyspepsia-related quality of life; FRDO, food retention disorder; LSDH, liver–stomach disharmony; SDQS, spleen deficiency with qi stagnation; SDS, single dyspepsia symptoms; SSDC, spleen and stomach deficiency and cold; STPI-FD, standard tool of pattern identification for functional dyspepsia; TACH, tangled cold and heat; TDS, total dyspepsia symptoms; VAS, visual analog scale. Correlation coefficients were analyzed using Pearson’s correlation analysis, where values between 0.6 and 0.8 indicate a high positive correlation and are highlighted in bold. Statistically significant results were adjusted using the Bonferroni correction (** *p* < 0.00125 and *** *p* < 0.000125).

## Data Availability

The data supporting the findings of this study are available from the corresponding author upon request.

## Supplementary Materials

The following supporting information can be downloaded at: https://www.mdpi.com/article/10.3390/healthcare12232331/s1, Supplement S1. The list of included literature following the full-text review, Refs. [51,52,53,54,55,56,57,58,59,60,61,62,63,64,65,66,67,68,69,70,71,72,73,74,75,76,77,78,79,80,81,82,83,84,85,86,87,88,89,90,91,92,93,94,95,96,97,98,99,100,101,102,103,104,105,106,107,108,109,110,111,112,113,114,115,116,117,118,119,120,121,122,123,124,125,126,127,128,129,130,131,132,133,134,135,136,137,138,139,140,141]; Supplement S2. Template of the final version of the standard pattern-identification tool for functional dyspepsia; Supplement S3. Final version of score-calculation formula for each pattern identification item.

## References

[1] Tack J., Talley N.J., Camilleri M., Holtmann G., Hu P., Malagelada J.R., Stanghellini V. (2006). Functional gastroduodenal disorders. Gastroenterology.

[2] Mahadeva S., Goh K.L. (2006). Epidemiology of functional dyspepsia: A global perspective. World J. Gastroenterol..

[3] Filipović B.F., Randjelovic T., Ille T., Markovic O., Milovanović B., Kovacevic N., Filipović B.R. (2013). Anxiety, personality traits and quality of life in functional dyspepsia-suffering patients. Eur. J. Intern. Med..

[4] Lacy B.E., Weiser K.T., Kennedy A.T., Crowell M.D., Talley N.J. (2013). Functional dyspepsia: The economic impact to patients. Aliment. Pharmacol. Ther..

[5] Sarnelli G., Caenepeel P., Geypens B., Janssens J., Tack J. (2003). Symptoms associated with impaired gastric emptying of solids and liquids in functional dyspepsia. Am. J. Gastroenterol..

[6] Tack J., Piessevaux H., Coulie B., Caenepeel P., Janssens J. (1998). Role of impaired gastric accommodation to a meal in functional dyspepsia. Gastroenterology.

[7] Tack J., Caenepeel P., Fischler B., Piessevaux H., Janssens J. (2001). Symptoms associated with hypersensitivity to gastric distention in functional dyspepsia. Gastroenterology.

[8] Mearin F., Cucala M., Azpiroz F., Malagelada J.R. (1991). The origin of symptoms on the brain-gut axis in functional dyspepsia. Gastroenterology.

[9] Lacy B.E., Talley N.J., Locke G.R., Bouras E.P., DiBaise J.K., El-Serag H.B., Abraham B.P., Howden C.W., Moayyedi P., Prather C. (2012). Review article: Current treatment options and management of functional dyspepsia. Aliment. Pharmacol. Ther..

[10] Stanghellini V., Chan F.K., Hasler W.L., Malagelada J.R., Suzuki H., Tack J., Talley N.J. (2016). Gastroduodenal disorders. Gastroenterology.

[11] Jiang M., Lu C., Zhang C., Yang J., Tan Y., Lu A., Chan K. (2012). Syndrome differentiation in modern research of traditional Chinese medicine. J. Ethnopharmacol.

[12] Ryu H.H., Lee H.J., Jang E.S., Choi S.M., Lee S.G., Lee S.W. (2008). Study on development of cold-heat pattern questionnaire. J. Physiol. Pathol. Korean Med..

[13] Ryu H.H., Lee H.J., Jang E.S., Lee S.W., Lee G.S., Kim J.Y. (2009). Study on deficiency-excess pattern questionnaire development possibility. J. Physiol. Pathol. Korean Med..

[14] Park Y.J., Yang D.H., Lee J.M., Park Y.B. (2013). Development of a valid and reliable blood stasis questionnaire and its relationship to heart rate variability. Complement. Ther. Med..

[15] Park J.S., Yang D.H., Kim M.Y., Lee S.C., Park Y.J., Park Y.B. (2006). Development of questionnaire for Damum patternization. J. Soc. Korean Med. Diagn..

[16] Park Y.J., Lim J.S., Park Y.B. (2013). Development of a valid and reliable food retention questionnaire. Eur. J. Integr. Med..

[17] Oh H.W., Lee J.W., Kim J.S., Song E.Y., Shin S.W., Han G.J., Lu H., Lee J.H. (2014). Study on the Development of a standard instrument of diagnosis and assessment for spleen qi deficiency pattern. J. Korean. Med..

[18] Lee J., Park J.W., Ko S.J., Kim J. (2018). Development and validation of a new pattern identification scale for Stomach Qi Deficiency. Eur. J. Integr. Med..

[19] Zhang S.S., Wang H.B., Li Q.J. (2002). Traditional Chinese medicine diagnostic and treatment guidelines for functional dyspepsia (draft). Chin. J. Integr. Tradit. West. Med. Dig..

[20] Han G.J., Kim J.S., Park J.W., Ryu B.H. (2011). Pattern identification of 97 functional dyspepsia patients and the characteristics of each pattern type. J. Korean. Med..

[21] Kim J.B., Kim J.H., Son C.G., Kang W.C., Cho J.H. (2010). Development of instrument of pattern identification for functional dyspepsia. J. Physiol. Pathol. Korean Med..

[22] Page M.J., McKenzie J.E., Bossuyt P.M., Boutron I., Hoffmann T.C., Mulrow C.D., Shamseer L., Tetzlaff J.M., Akl E.A., Brennan S.E. (2021). The PRISMA 2020 statement: An updated guideline for reporting systematic reviews. BMJ.

[23] Han G.J., Leem J.T., Lee N.L., Kim J.S., Park J.W., Lee J.H. (2015). Development of a standard tool for pattern identification of gastroesophageal reflux disease (GERD). J. Intern. Korean Med..

[24] Zhang S., Zhao L., Wang H., Wang C., Huang S., Shen H., Wei W., Tao L., Zhou T. (2013). Efficacy of modified LiuJunZi decoction on functional dyspepsia of spleen-deficiency and qi-stagnation syndrome: A randomized controlled trial. BMC Complement. Altern. Med..

[25] Lee E.H., Hahm K.B., Lee J.H., Park J.J., Lee D.H., Kim S.K., Choi S.R., Lee S.T. (2006). Development and validation of a functional dyspepsia-related quality of life (FD-QOL) scale in South Korea. J. Gastroenterol. Hepatol..

[26] Tavakol M., Dennick R. (2011). Making sense of Cronbach’s alpha. Int. J. Med. Educ..

[27] Zha Q.L., He Y.T., Yu J.P. (2006). Correlations between diagnostic information and therapeutic efficacy in rheumatoid arthritis analyzed with decision tree model. Chin. J. Integr. Tradit. West. Med..

[28] Mist S., Ritenbaugh C., Aickin M. (2009). Effects of questionnaire-based diagnosis and training on inter-rater reliability among practitioners of traditional Chinese medicine. J. Altern. Complement. Med..

[29] Byun J.S., Yang S.Y., Kang W.C., Park Y.C. (2009). Primary study to develop the instrument of pattern identification for common cold. J. Physiol. Pathol. Korean Med..

[30] Park E.J., Jang S.B., Baek S.E., Kim S.K., Yoo H.R., Yoo J.E., Jung I.C. (2017). Preliminary study on development of pattern identification instrument for polycystic ovarian syndrome. J. Korean Obstet. Gynecol..

[31] Lee E.H., Choi W.C., Jung I.C. (2013). Preliminary study to develop the instrument on pattern identifications for depression. J. Orient. Neuropsychiatry.

[32] Wei Y.M., Lee G.E., Jung S.H., Lee H.K., Lyu Y.S., Kang H.W. (2012). A Study on the Reliability and Factor analysis of Pattern Identification for Tic Disorders in children. J. Orient. Neuropsychiatry.

[33] Kim K.K., Seo B.N., Kang W.C., Jung I.C. (2013). Guidelines for the reliability and validity of the instrument on pattern identifications for Hwa-byung. J. Orient. Neuropsychiatry.

[34] Kim J., Kim J., Kim J., Kim K.H. (2015). Reliability and validity analysis of a standard instrument of diagnosis and assessment for spleen Qi deficiency pattern in chronic dyspepsia patients. J. Korean Med..

[35] Nam S., Park J., Kim J. (2018). Correlation analysis between stomach qi deficiency pattern and gastric emptying measured by abdominal ultrasonography in patients with functional dyspepsia. J. Intern. Korean Med..

[36] Hwang J.H., Ko D. (2018). Literature review of clinical studies for the relationship between ultrasonographic examination and syndrome differentiation classification in Chinese medicine. J. Physiol. Pathol. Korean Med..

[37] Ha N.Y., Ko S.J., Park J.W., Kim J. (2023). Efficacy and safety of the herbal formula Naesohwajung-tang for functional dyspepsia: A randomized, double-blind, placebo-controlled, multi-center trial. Front. Pharmacol..

[38] Li D.D., Yue Z.H., Xu L.C., Xie T., Hu G.Z., Yang J. (2014). Clinical evaluation study on long-term effect of acupuncture with pattern/syndrome differentiation on functional dyspepsia. Zhounguo Zhen Jiu.

[39] Chen P., Chen A.P. (2020). Clinical observation of “experienced ten acupoints” for functional dyspepsia of liver stagnation and spleen deficiency. Zhongguo Zhen Jiu.

[40] Taherdoost H. (2016). Validity and Reliability of the Research Instrument; How to Test the Validation of a Questionnaire/Survey in a Research. Int. J. Acad. Res. Manag..

[41] Bujang M.A., Omar E.D., Baharum N.A. (2018). A review on sample size determination for Cronbach’s alpha test: A simple guide for researchers. Malays. J. Med. Sci..

[42] Kang H. (2013). A guide on the use of factor analysis in the assessment of construct validity. J. Korean. Acad. Nurs..

[43] Tally N.J., Verlinden M., Jone M. (2001). Quality of life in functional dyspepsia: Responsiveness of the Nepean Dyspepsia Index and development of a new 10-item short form. Aliment. Pharmacol. Ther..

[44] Lee B., Ha N.Y., Park H.J., Kim A.R., Kwon O.J., Cho J.H., Shin S.M., Kim J., Yang C. (2023). Herbal Medicine Yukgunja-Tang for Functional Dyspepsia: A Protocol for a Randomized, Controlled, Multicenter Clinical Trial. Healthcare.

[45] Ko S.J., Park J.W., Lee J.H., Lee J.E., Ha N.Y., Nam S.U., Lee J.H., Jeon S.H., Kim J.W., Kang C. (2018). An Herbal Medicine, Yukgunja-Tang is more Effective in a Type of Functional Dyspepsia Categorized by Facial Shape Diagnosis: A Placebo-Controlled, Double-Blind, Randomized Trial. Evid. Based. Complement. Alternat. Med..

[46] Altman D.G. (1990). Practical Statistics for Medical Research.

[47] Lacy B.E., Everhart K., Crowell M.D. (2011). Functional dyspepsia is associated with sleep disorders. Clin. Gastroenterol. Hepatol..

[48] Cremonini F., Talley N.J. (2004). Review article: The overlap between functional dyspepsia and irritable bowel syndrome—A tale of one or two disorders?. Aliment. Pharmacol. Ther..

[49] Oudenhove L.V., Vandenberghe J., Vos R., Holvoet L., Tack J. (2011). Factors associated with co-morbid irritable bowel syndrome and chronic fatigue-like symptoms in functional dyspepsia. Neurogastroenterol. Motil..

[50] Charter R.A. (1999). Sample size requirements for precise estimates of reliability, generalizability, and validity coefficients. J. Clin. Exp. Neuropsychol..

[51] Son J.Y., Kim J.S. (2014). Diagnostic values of tongue coating thickness and sterno-costal angle in functional dyspepsia. J. Int. Korean. Med..

[52] Kim Y.M., Park Y.C., Jo J.H., Kang W.C., Son W.M., Hong K.E. (2010). Effect of herb medicine treatment for functional dyspepsia: A randomized placebo-controlled and compared standard treatment trial. J. Korean Med..

[53] Lee J.J., Son M.W., Hong K.E. (2009). Effect of herb drug medicine treatment for functional dyspepsia: Controlled trial. J. Pharmacoacupunct..

[54] Park Y.C., Cho J.H., Choi S.M., Son C.G. (2008). Analytic study of 68 patients with functional dyspepsia according to syndrome differentiation. J. Int. Korean Med..

[55] Kim D.W., Choi B.H., Hur J.I., Park K., Kim D.J., Byun J.S. (2006). Evaluation for therapeutic effectiveness of bowhatang in functional dyspepsia. Herb. Formula Sci..

[56] Kim H.K., Yoon S.H., Lee J.S., Eom G.H., Lee S.Y., Kim S.Y., Hur W.Y., Kim J.S., Ryu B.H. (2006). Correlation study between fatigue degree and comprehensive diagnosis of Qui Xui Shui in patients with functional dyspepsia. J. Int. Korean Med..

[57] Oh J.H., Kim B.S., Lim H.Y., Kim D.W., Choi B.H., Hur J.I., Kim D.J., Cho C.K., Byun J.S. (2005). Three cases report of functional dyspepsia patients who were administered by LJTG(Lijintang-Gamibang). J. Int. Korean Med..

[58] Han S.Y., Lim J.H., Ryu J.M., Jang S.Y., Kim H.K., Lee J.S., Yoon S.H., Kim J.S., Ryu B.H., Ryu K.W. (2004). Analysis of symptom pattern through comprehensive diagnosis of Qui Xui Shui in patients with functional dyspepsia. J. Int. Korean Med..

[59] Jeong H.D., Yoon S.H., Kim J.S., Ryu B.H., Ryu K.W. (2004). Relationship between gastric motility and health condition graded by total symptom scores in comprehensive diagnosis of Qui Xue Shui in functional dyspeptic patients. J. Int. Korean Med..

[60] Hong S.H. (2014). Research on Korean Medicine Doctors’ Decision-Making on Diagnosis and Selection of Acupoints. Master’s Thesis.

[61] Department of Diagnosis Disease National Coalition Korean Medicine (2008). Digestive Diseases.

[62] Zhang X. (2018). Clinical Observation on of Traditional Chinese Medicine Syndromes Differentiation in Treating Functional Dyspepsia. Guangming J. Chin. Med..

[63] Zhu W. (2018). Study on TCM Clinical Syndrome Differentiation of Functional Dyspepsia. Chin. Health Stand. Manag..

[64] Gou X., Wu W., Gao N., Wang W. (2018). Treatment of functional dyspepsia with Traditional Chinese Medicine syndrome differentiation and modifications of classical prescriptions. Cardiovasc. Dis. J. Integr. Tradit. Chin. West. Med..

[65] Peng W. (2017). Clinical Study on 60 Cases of Functional Dyspepsia Dialectical Treated by Modified Tiaowei Jianpi Soup. Master’s Thesis.

[66] Cai J. (2017). Clinical effect of TCM syndrome differentiation on spleen and stomach type of functional dyspepsia. Chin. J. Clin. Rational Drug Use..

[67] Wang W. (2017). Study on the application effect of Traditional Chinese Medicine syndrome differentiation and treatment in patients with spleen-stomach qi deficiency type functional dyspepsia. J. Clin. Med. Lit..

[68] Fu D. (2017). Clinical efficacy of Traditional Chinese Medicine syndrome differentiation in the treatment of spleen-stomach qi deficiency type functional dyspepsia. World Latest Med. Inf..

[69] Ye A. (2016). Methods and Clinical Efficacy of TCM Syndrome Differentiation Treatment for Spleen and Stomach Deficiency Type Functional Dyspepsia. Nei Mongol J. Tradit. Chin. Med..

[70] Ge X. (2016). Clinical observation of functional dyspepsia treated by syndrome differentiation in Traditional Chinese Medicine. J. New Chin. Med..

[71] Li J. (2016). Clinical Experience in TCM Syndrome Differentiation and Treatment of 82 Cases of Functional Dyspepsia. Nei Mongol J. Tradit. Chin. Med..

[72] Deng M., Wang L., Jin L.P., Sun S.Y., Ren Q.L. (2016). Spleen Qi Deficiency Functional Dyspepsia Efficacy of TCM Treatment. China Foreign Med. Treat..

[73] Wang R., Yang Y. (2016). Clinical study on the treatment of functional dyspepsia with syndrome differentiation in Traditional Chinese Medicine. Asia-Pac. Tradit. Med..

[74] Zhong H. (2016). Observation on the efficacy of treating spleen-stomach qi deficiency type functional dyspepsia with syndrome differentiation in Traditional Chinese Medicine. Cap. Food Med..

[75] Zhang S.S., Zhao L.Q., Wang C.J., Shen H., Huang S.P., Wei W., Wang H.B., Wu B., Li Y.F., Liu Y.J. (2016). Efficacy of syndrome differentiation based on ’han,re,xu,shi’ on functional dyspepsia: A randomized controlled patient-reported trial. Chin. J. Tradit. Chin. Med. Pharm..

[76] Wu Y. (2015). Evaluation of the effect of syndrome differentiation in Traditional Chinese Medicine for treating functional dyspepsia. For All Health.

[77] Liu Y., Shen H., Cui Y., Li H., Ge C., Xu Y. (2015). Clinical Study on Syndrome Differentiation Treatment of 50 Cases of Functional Dyspepsia. Jiangsu J. Tradit. Chin. Med..

[78] Guo X., Ren L., Lin H., Zhang K., Jiang K., Fu S. (2015). Study of Acupuncture on Liver Stagnation Type of Functional Dyspepsia and Its Metabolomics. Liaoning J. Tradit. Chin. Med..

[79] Xiang Y., Chen S., Qing X. (2015). Observation on the Efficacy of TCM Syndrome Differentiation and Treatment of Spleen and Stomach Qi Deficiency Type Functional Dyspepsia. For All Health.

[80] Chen H. (2015). Clinical Observation on Functional Dyspepsia Treated by Acupuncture on Syndrome Differentiation and Investigatation 5- HT of FD Rats with Disharmony Between Liver and Stomach Syndrome. Master’s Thesis.

[81] Feng Y. (2015). Study on the Clinical Efficacy of TCM Differentiation and Treatment of Functional Dyspepsia. For All Health.

[82] Wang Y.Y., Li J., Zhang F.B., Zhang R.X. (2015). The relationship between the subtypes of functional dyspepsia and TCM differentiation and the level of GLP-1. Chin. J. Integr. Tradit. West. Med. Dig..

[83] Liu J., Li F., Tang X., Ma J., Bai S., Liu Y. (2015). Modern Clinical Studies on TCM Syndrome of Functional Dyspepsia and their Differentiation Standards. World Chin. Med..

[84] Ye Y. (2014). A Randomized Controlled Study of Traditional Chinese Medicine Syndrome Differentiation and Treatment of Functional Dyspepsia. J. Pract. Tradit. Chin. Intern. Med..

[85] Lin Y., Chen X. (2014). Syndrome Differentiation and Treatment of 124 Cases of Functional Dyspepsia. China Foreign Med. Treat..

[86] Xia P. (2014). Clinical Study on the Efficacy of TCM Syndrome Differentiation and Treatment of Spleen and Stomach Qi Deficiency Type Functional Dyspepsia. Contemp. Med. Forum.

[87] Zeng J. (2014). Clinical Observation of 52 Cases of Functional Dyspepsia Treated by Syndrome Differentiation. J. Med. Theory Pract..

[88] Huang Q. (2014). The Effect on Living Quality, Serum Gastirn and Motilin of Functional Dyspepsia Patients Treated with Acupuncture Based on Syndrome Differentiation. Master’s Thesis.

[89] Li Q., Xiao Z., Chen F. (2014). TCM Syndrome Differentiation and Treatment of 81 Cases of Functional Dyspepsia. Fujian J. Tradit. Chin. Med..

[90] Liu D. (2014). Clinical Observation on Syndrome Differentiation Treatment of 124 Cases of Functional Dyspepsia. Res. Integr. Tradit. Chin. West. Med..

[91] Yang S. (2014). Clinical Observation of the Efficacy of TCM Syndrome Differentiation Treatment of Functional Dyspepsia. Guide China Med..

[92] Xu W.H., Yao S.K., Li N.J., Zhang Y.L., Ke M.Y. (2013). Influences of TCM syndrome differentiation and treatment on anxiety and depression status in patients with functional dyspepsia. J. Beijing Univ. Tradit. Chin. Med..

[93] Xu W.H., Yao S.K., Li N.J., Zhang Y.L., Ke M.Y., Wang X.Y. (2013). Study of TCM differentiating treatment on patients with functional dyspepsia. Chin. J. Integr. Tradit. West. Med. Dig..

[94] Jin L., Hu Y., Gao Z., Zhou L., Hou L., Zhang W., Zhang H. (2013). Clinical Curative Effect Evaluation of Acupuncture by Syndrome Differentiation for Functional Dyspepsia. Liaoning J. Tradit. Chin. Med..

[95] Zhang F. (2013). TCM Syndrome Differentiation Treatment of Functional Dyspepsia. Mod. Diagn. Treat..

[96] Liu Y. (2013). Observation on the Efficacy of Syndrome Differentiation Treatment and Care for Functional Dyspepsia. Clin. J. Tradit. Chin. Med..

[97] Cai S., Wang X. (2013). Syndrome Differentiation Treatment of Functional Dyspepsia. Hebei J. Tradit. Chin. Med..

[98] Chen R. (2013). Comparative Study of TCM Syndrome Differentiation and Western Medicine Classification in Functional Dyspepsia. Master’s Thesis.

[99] Zhou H. (2013). Experience in TCM Syndrome Differentiation and Treatment of 39 Cases of Functional Dyspepsia. Chin. J. Ethnomed. Ethnopharm..

[100] Men J. (2013). Syndrome Differentiation Treatment of 41 Cases of Functional Dyspepsia. Zhejiang J. Tradit. Chin. Med..

[101] Yang D. (2012). Analysis of TCM Syndrome Differentiation Treatment of Functional Dyspepsia. China J. Pharm. Econ..

[102] Liu Q.M. (2012). Clinical Observation of Treatment Based on Syndrome Differentiation Acupuncture on Functional Dyspepsia with Sleep Disorders. Master’s Thesis.

[103] Hu Y. (2012). Therapeutic Effect Observation on Functional Dyspepsia Treated with Acupuncture by Differentiation of Symptoms and Signs and its Effect on Serum Gastrin. Master’s Thesis.

[104] Cui Y. (2012). Evaluation of Clinical Curative Effect on Treatment of Functional Dyspepsia Based on Syndrome Differentiation with Excess and Deficiency Pattern. Master’s Thesis.

[105] Wang Y. (2012). The Relationship between the Subtypes of Functional Dyspepsia and TCM Differentiation and the Level of GLP-1. Master’s Thesis.

[106] Liu G., Lin P. Exploration and Comparison of the Essential Pathogenesis of Functional Dyspepsia Using Element and Organ Syndrome Differentiation. Proceedings of the 24th National Conference of Spleen Stomach Disease Chinese Association Chinese Traditional Medicine.

[107] Bi W. (2012). Syndrome Differentiation Treatment of 50 Cases of Functional Dyspepsia with Tongjiang Weikang Decoction. Chin. Med. Mod. Dist. Educ. China.

[108] Liu H. (2011). Syndrome Differentiation Treatment of 60 Cases of Functional Dyspepsia. Shaanxi J. Tradit. Chin. Med..

[109] Lei X. (2011). The Study of Syndrome Differentiation in Traditional Chinese Medicine of Functional Dyspepsia and the Relationship between LEP and PYY. Master’s Thesis.

[110] Lu H. (2011). Efficacy Observation of TCM Syndrome Differentiation Treatment of Spleen and Stomach Qi Deficiency Type Functional Dyspepsia. China Foreign. Med. Treat..

[111] Wang J. (2010). TCM Syndrome Differentiation and Treatment of Functional Dyspepsia. Gansu J. Tradit. Chin. Med..

[112] Su G. (2010). Current Status of Research on TCM Syndrome Differentiation Treatment of Functional Dyspepsia. China Mod. Doctor.

[113] Hua Z., Cai H. (2010). Observation on the Efficacy of Syndrome Differentiation Treatment of 45 Cases of Functional Dyspepsia. Shandong J. Tradit. Chin. Med..

[114] Chen L. Syndrome Differentiation and Treatment Characteristics of Functional Dyspepsia. Proceedings of the 22nd National Symposium Integrated Traditional Chinese Western Medicine Digest System Disease.

[115] Cao D., Yang Z. (2010). Analysis of 87 Cases of Functional Dyspepsia Treated by TCM Syndrome Differentiation and Classification. Aerospace Med..

[116] Pan F. (2010). Syndrome Differentiation and Classification Treatment of Functional Dyspepsia. Mod. J. Integr. Tradit. Chin. West. Med..

[117] Li C.Y., Wu H.Z., Ou H.J., Qiu Q. (2010). Clinical Observation of Elderly Patient’s Functional Dyspepsia Treated with Dialectic. Liaoning J. Tradit. Chin. Med..

[118] Su G. (2010). Observation on the Efficacy of Syndrome Differentiation Treatment of 40 Cases of Functional Dyspepsia. Nei Mongol J. Tradit. Chin. Med..

[119] Zhang F. (2010). Analyzing TCM Syndromes of Functional Dyspepsia and the Relationship between Various Involving Factors. Master’s Thesis.

[120] Guo X. (2010). Distribution Rule of Syndrome Differentiation in Traditional Chinese Medicine in Functional Dyspepsia. Master’s Thesis.

[121] Zeng C. (2010). Thoughts on TCM Syndrome Differentiation and Treatment of Functional Dyspepsia. Chin. J. Ethnomed. Ethnopharm..

[122] Wei W., Shi H.X., Fan L.N. (2009). The relation between functional dyspepsia’s Rome Ⅲ criteria and traditional Chinese medicine syndrome. Glob. Tradit. Chin. Med..

[123] Zhang B. (2009). Efficacy Observation of TCM Syndrome Differentiation Treatment of 54 Cases of Spleen and Stomach Qi Deficiency Type Functional Dyspepsia. Heilongjiang J. Tradit. Chin. Med..

[124] Zheng Z. (2009). Summary of Experience in TCM Syndrome Differentiation Treatment of Functional Dyspepsia. Chin. J. Ethnomed. Ethnopharm..

[125] Cai F. (2009). Efficacy Observation of TCM Syndrome Differentiation Treatment of 120 Cases of Spleen and Stomach Qi Deficiency Type Functional Dyspepsia. Chin. J. Mod. Drug. Appl..

[126] Xue L., Zhang H. (2008). Syndrome Differentiation and Classification Treatment of 60 Cases of Functional Dyspepsia. J. Liaoning Univ. Tradit. Chin. Med..

[127] Guan S. (2008). Analysis of Syndrome Differentiation and Treatment of Functional Dyspepsia. J. Med. Forum.

[128] Li Y., Wei Y. (2008). TCM Syndrome Differentiation Treatment of Functional Dyspepsia. Chin. Med. Mod. Dist. Educ. China.

[129] Liu S. (2008). Syndrome Differentiation and Treatment of Functional Dyspepsia. Public Med. Forum Mag..

[130] Zhao L. (2007). TCM Syndrome Differentiation Treatment of Functional Dyspepsia. Guangming J. Chin. Med..

[131] Zhang S. (2007). Syndrome Differentiation and Classification Treatment of 32 Cases of Refractory Functional Dyspepsia. J. Pract. Med..

[132] Xi D. (2007). Clinical Observation of 52 Cases of Functional Dyspepsia Treated by Syndrome Differentiation. Forum Tradit. Chin. Med..

[133] Zhao X. (2006). Syndrome Differentiation and Classification Treatment of 40 Cases of Functional Dyspepsia. Shaanxi J. Tradit. Chin. Med..

[134] Li G. (2006). Syndrome Differentiation and Treatment of 76 Cases of Functional Dyspepsia. Tradit. Chin. Med. Res..

[135] Fan H. (2006). Summary of 45 Cases of Functional Dyspepsia Treated by Syndrome Differentiation and Classification. J. Sichuan Tradit. Chin. Med..

[136] Wang Y. (2006). Syndrome Differentiation and Classification Treatment of 86 Cases of Functional Dyspepsia. J. Sichuan Tradit. Chin. Med..

[137] Liu S. (2006). TCM Syndrome Differentiation and Treatment of Functional Dyspepsia. J. Pract. Med. Tech..

[138] Gu C., Zhang L., Li W. (2006). Clinical Research on Syndrome Differentiation and Classification Treatment of Functional Dyspepsia. Chin. Arch. Tradit. Chin. Med..

[139] Ye P. (2006). Observation on the Efficacy of Syndrome Differentiation Treatment of Functional Dyspepsia. Chin. J. Rural Med. Pharm..

[140] Wang J., Zhou W. (2005). Analysis of 51 Cases of Functional Dyspepsia Treated by Syndrome Differentiation. J. Pract. Tradit. Chin. Intern. Med..

[141] An S. (2005). Analysis of 80 Cases of Functional Dyspepsia Treated by Syndrome Differentiation. J. Pract. Tradit. Chin. Intern. Med..

