# Supplementary material for: Development of a Standard Tool of Pattern Identification for Functional Dyspepsia: A Cross-Sectional Study from Korea
Source: Healthcare (Basel). 2024 Nov 21;12(23):2331. doi: 10.3390/healthcare12232331 (PMC11641813; doi:10.3390/healthcare12232331)
Supplement: Supplementary file 1 [file healthcare-12-02331-s001.zip › healthcare-3289764-supplementary.pdf]

Supplement S1. The list of included literature following the full-text review.

1) Korean (n = 13)

1. Han, G.J.; Kim, J.S.; Park, J.W.; Ryu, B.H. Pattern identification of 97 functional dyspepsia patients and the characteristics of each pattern type. *J. Korean. Med.* **2011**, *32*, 42–62.
2. Kim, J.B.; Kim, J.H.; Son, C.G.; Kang, W.C.; Cho, J.H. Development of instrument of pattern identification for functional dyspepsia. *J. Physiol. Pathol. Korean Med*. **2010**, *24*, 1094–1098.
3. Son, J.Y.; Kim, J.S. Diagnostic values of tongue coating thickness and sterno-costal angle in functional dyspepsia. *J. Int. Korean. Med*. **2014**, *35*, 157-174.
4. Kim, Y.M.; Park, Y.C.; Jo, J.H.; Kang, W.C.; Son, W.M.; Hong, K.E. Effect of herb medicine treatment for functional dyspepsia : a randomized placebo-controlled and compared standard treatment trial. *J. Korean Med*. **2010**, *31*, 1-13.
5. Lee J.J.; Son, M.W.; Hong, K.E. Effect of herb drug medicine treatment for functional dyspepsia: controlled trial. *J. Pharmacoacupunct*. **2009**, *12*, 51-66.
6. Park, Y.C.; Cho, J.H.; Choi, S.M.; Son, C.G. Analytic study of 68 patients with functional dyspepsia according to syndrome differentiation. *J. Int. Korean Med*. **2008**, *29*, 574-581.
7. Kim, D.W.; Choi, B.H.; Hur, J.I.; Park, K.; Kim, D.J.; Byun, J.S. Evaluation for therapeutic effectiveness of bowhatang in functional dyspepsia. *Herb. Formula Sci*. **2006**, *14*, 97-108.
8. Kim, H.K.; Yoon, S.H.; Lee, J.S.; Eom, G.H.; Lee, S.Y.; Kim, S.Y.; Hur, W.Y.; Kim, J.S.; Ryu, B.H. Correlation study between fatigue degree and comprehensive diagnosis of Qui Xui Shui in patients with functional dyspepsia. *J. Int. Korean Med*. **2006**, *27*, 510-520.
9. Oh, J.H.; Kim, B.S.; Lim, H.Y.; Kim, D.W.; Choi, B.H.; Hur, J.I.; Kim, D.J.; Cho, C.K.; Byun, J.S. Three cases report of functional dyspepsia patients who were administered by LJTG(Lijintang-Gamibang). *J. Int. Korean Med*. **2005**, *26*, 641-651.
10. Han, S.Y.; Lim, J.H.; Ryu, J.M.; Jang, S.Y.; Kim, H.K.; Lee, J.S.; Yoon, S.H.; Kim, J.S.; Ryu, B.H.; Ryu, K.W. Analysis of symptom pattern through comprehensive diagnosis of Qui Xui Shui in patients with functional dyspepsia. *J. Int. Korean Med*. **2004**, *25*, 224-237.
11. Jeong, H.D.; Yoon, S.H.; Kim, J.S.; Ryu, B.H.; Ryu, K.W. Relationship between gastric motility and health condition graded by total symptom scores in comprehensive diagnosis of Qui Xue Shui in functional dyspeptic patients. *J. Int. Korean Med*. **2004**, *25*, 158-166.
12. Hong, S.H. *Research on Korean medicine doctors' decision-making on diagnosis and selection of acupoints*; MA thesis: Kyung Hee Univ., Seoul, Republic of Korea, 2014.
13. Dept. Dig. Dis. Nat'l. Coll. Korean Med. *Digestive Diseases*, 1st ed.; Koonja: Seoul, Republic of Korea, 2008.

2) Chinese (n = 80)

1. Zhang, X. Clinical Observation on of Traditional Chinese Medicine Syndromes Differentiation in Treating Functional Dyspepsia. *Guangming J. Chin. Med.* **2018**, 33, 955-957.
2. Zhu, W. Study on TCM Clinical Syndrome Differentiation of Functional Dyspepsia. *Chin. Health Stand. Manag.* **2018**, 9, 108-109.
3. Gou, X.; Wu, W; Gao, N; Wang W. Treatment of functional dyspepsia with Traditional Chinese Medicine syndrome differentiation and modifications of classical prescriptions. *Cardiovasc. Dis. J. Integr. Tradit. Chin. West. Med.* **2018**, 6, 135-138.
4. Peng, W. Clinical Study on 60 Cases of Functional Dyspepsia Dialectical Treated by Modified Tiaowei Jianpi Soup. MA thesis: Hubei Univ. Chin. Med., Wuhan, People's Republic of China, **2017**.
5. Cai, J. Clinical effect of TCM syndrome differentiation on spleen and stomach type of functional dyspepsia. *Chin. J. Clin. Rational Drug Use.* **2017**, 10, 30-31.
6. Wang, W. Study on the application effect of Traditional Chinese Medicine syndrome differentiation

and treatment in patients with spleen-stomach qi deficiency type functional dyspepsia. *J. Clin. Med. Lit.* **2017**, 4, 8353-8354.
7. Fu, D. Clinical efficacy of Traditional Chinese Medicine syndrome differentiation in the treatment of spleen-stomach qi deficiency type functional dyspepsia. *World Latest Med. Inf.* **2017**, 17, 172-173.
8. Ye, A. Methods and Clinical Efficacy of TCM Syndrome Differentiation Treatment for Spleen and Stomach Deficiency Type Functional Dyspepsia. *Nei Mongol J. Tradit. Chin. Med.* **2016**, 35, 35.
9. Ge, X. Clinical observation of functional dyspepsia treated by syndrome differentiation in Traditional Chinese Medicine. *J. New Chin. Med.* **2016**, 48, 46-47.
10. Li, J. Clinical Experience in TCM Syndrome Differentiation and Treatment of 82 Cases of Functional Dyspepsia. *Nei Mongol J. Tradit. Chin. Med.* **2016**, 35, 58.
11. Deng, M.; Wang, L.; Jin, L.P.; Sun, S.Y.; Ren, Q.L. Spleen Qi Deficiency Functional Dyspepsia Efficacy of TCM Treatment. *China Foreign Med. Treat.* **2016**, 35, 167-168.
12. Wang, R.; Yang, Y. Clinical study on the treatment of functional dyspepsia with syndrome differentiation in Traditional Chinese Medicine. *Asia-Pac. Tradit. Med.* **2016**, 12, 106-107.
13. Zhong, H. Observation on the efficacy of treating spleen-stomach qi deficiency type functional dyspepsia with syndrome differentiation in Traditional Chinese Medicine. *Cap. Food Med.* **2016**, 23, 65-66.
14. Zhang, S.S.; Zhao, L.Q.; Wang, C.J.; Shen, H.; Huang, S.P.; Wei, W.; Wang, H.B.; Wu, B.; Li, Y.F.; Liu, Y.J.; Huang, S.G.; Lai, Y.L. Efficacy of syndrome differentiation based on 'han,re,xu,shi' on functional dyspepsia:A randomized controlled patient-reported trial. *Chin. J. Tradit. Chin. Med. Pharm.* **2016**, 31, 65-71.
15. Wu, Y. Evaluation of the effect of syndrome differentiation in Traditional Chinese Medicine for treating functional dyspepsia. *For All Health* **2015**, 9, 36-37.
16. Liu, Y.; Shen, H.; Cui, Y.; Li, H.; Ge, C.; Xu, Y. Clinical Study on Syndrome Differentiation Treatment of 50 Cases of Functional Dyspepsia. *Jiangsu J. Tradit. Chin. Med.* **2015**, 47, 41-43.
17. Guo, X.; Ren, L.; Lin, H.; Zhang, K.; Jiang, K.; Fu, S. Study of Acupuncture on Liver Stagnation Type of Functional Dyspepsia and Its Metabolomics. *Liaoning J. Tradit. Chin. Med.* **2015**, 42, 1752-1754.
18. Xiang, Y.; Chen, S.; Qing, X. Observation on the Efficacy of TCM Syndrome Differentiation and Treatment of Spleen and Stomach Qi Deficiency Type Functional Dyspepsia. *For All Health* **2015**, 9, 33-34.
19. Chen, H. Clinical Observation on Functional Dyspepsia Treated by Acupuncture on Syndrome Differentiation and Investigatation 5- HT of FD Rats with Disharmony Between Liver and Stomach Syndrome. MA thesis: Hubei Univ. Chin. Med., Wuhan, People's Republic of China, **2015**.
20. Feng, Y. Study on the Clinical Efficacy of TCM Differentiation and Treatment of Functional Dyspepsia. *For All Health* **2015**, 9, 33.
21. Wang, Y.Y.; Li, J.; Zhang, F.B.; Zhang, R.X. The relationship between the subtypes of functional dyspepsia and TCM differentiation and the level of GLP-1. *Chin. J. Integr. Tradit. West. Med. Dig.* **2015**, 23, 112-117.
22. Liu, J.; Li, F.; Tang, X.; Ma, J.; Bai, S.; Liu, Y. Modern Clinical Studies on TCM Syndrome of Functional Dyspepsia and their Differentiation Standards. *World Chin. Med.* **2015**, 10, 56-59.
23. Ye, Y. A Randomized Controlled Study of Traditional Chinese Medicine Syndrome Differentiation and Treatment of Functional Dyspepsia. *J. Pract. Tradit. Chin. Intern. Med.* **2014**, 28, 104-106.
24. Lin, Y.; Chen, X. Syndrome Differentiation and Treatment of 124 Cases of Functional Dyspepsia. China Foreign Med. Treat. **2014**, 33, 153-154.
25. Xia, P. Clinical Study on the Efficacy of TCM Syndrome Differentiation and Treatment of Spleen and Stomach Qi Deficiency Type Functional Dyspepsia. *Contemp. Med. Forum* **2014**, 12, 156-157.
26. Zeng, J. Clinical Observation of 52 Cases of Functional Dyspepsia Treated by Syndrome Differentiation. *J. Med. Theory Pract.* **2014**, 27, 1453-1454.

27. Huang, Q. The Effect on Living Quality, Serum Gastirn and Motilin of Functional Dyspepsia Patients Treated with Acupuncture Based on Syndrome Differentiation. MA thesis: Hubei Univ. Chin. Med., Wuhan, People's Republic of China, **2014**.
28. Li, Q.; Xiao, Z.; Chen, F. TCM Syndrome Differentiation and Treatment of 81 Cases of Functional Dyspepsia. *Fujian J. Tradit. Chin. Med.* **2014**, 45, 32-33.
29. Liu, D. Clinical Observation on Syndrome Differentiation Treatment of 124 Cases of Functional Dyspepsia. *Res. Integr. Tradit. Chin. West. Med.* **2014**, 6, 154-155.
30. Yang, S. Clinical Observation of the Efficacy of TCM Syndrome Differentiation Treatment of Functional Dyspepsia. *Guide China Med.* **2014**, 12, 299.
31. Xu, W.H.; Yao, S.K.; Li, N.J.; Zhang, Y.L.; Ke, M.Y. Influences of TCM syndrome differentiation and treatment on anxiety and depression status in patients with functional dyspepsia. *J. Beijing Univ. Tradit. Chin. Med.* **2013**, 36, 640-644.
32. Xu, W.H.; Yao, S.K.; Li, N.J.; Zhang, Y.L.; Ke, M.Y.; Wang, X.Y. Study of TCM differentiating treatment on patients with functional dyspepsia. *Chin. J. Integr. Tradit. West. Med. Dig.* **2013**, 21, 225-228.
33. Jin, L.; Hu, Y.; Gao, Z.; Zhou, L.; Hou, L.; Zhang, W.; Zhang, H. Clinical Curative Effect Evaluation of Acupuncture by Syndrome Differentiation for Functional Dyspepsia. *Liaoning J. Tradit. Chin. Med.* **2013**, 40, 1222-1225.
34. Zhang, F. TCM Syndrome Differentiation Treatment of Functional Dyspepsia. *Mod. Diagn. Treat.* **2013**, 24, 1006-1007.
35. Liu, Y. Observation on the Efficacy of Syndrome Differentiation Treatment and Care for Functional Dyspepsia. *Clin. J. Tradit. Chin. Med.* **2013**, 25, 53-54.
36. Cai, S.; Wang, X. Syndrome Differentiation Treatment of Functional Dyspepsia. *Hebei J. Tradit. Chin. Med.* **2013**, 35, 1156-1233.
37. Chen, R. Comparative Study of TCM Syndrome Differentiation and Western Medicine Classification in Functional Dyspepsia. MA thesis: Liaoning Univ. Tradit. Chin. Med., Shenyang, People's Republic of China, **2013**.
38. Zhou, H. Experience in TCM Syndrome Differentiation and Treatment of 39 Cases of Functional Dyspepsia. *Chin. J. Ethnomed. Ethnopharm.* **2013**, 22, 110.
39. Men, J. Syndrome Differentiation Treatment of 41 Cases of Functional Dyspepsia. *Zhejiang J. Tradit. Chin. Med.* **2013**, 48, 179.
40. Yang, D. Analysis of TCM Syndrome Differentiation Treatment of Functional Dyspepsia. *China J. Pharm. Econ.* **2012**, 6, 195-196.
41. Liu, Q.M. Clinical Observation of Treatment Based on Syndrome Differentiation Acupuncture on Functional Dyspepsia with Sleep Disorders. MA thesis: Hubei Univ. Chin. Med., Wuhan, People's Republic of China, **2012**.
42. Hu, Y. Therapeutic Effect Observation on Functional Dyspepsia Treated with Acupuncture by Differentiation of Symptoms and Signs and its Effect on Serum Gastrin. MA thesis: Hubei Univ. Chin. Med., Wuhan, People's Republic of China, **2012**.
43. Cui, Y. Evaluation of Clinical Curative Effect on Treatment of Functional Dyspepsia Based on Syndrome Differentiation with Excess and Deficiency Pattern. MA thesis: Nanjing Univ. of Chin. Med., Nanjing, People's Republic of China, **2012**.
44. Wang, Y. The Relationship between the Subtypes of Functional Dyspepsia and TCM Differentiation and the Level of GLP-1. MA thesis: Heibei Med. Univ., Shijiazhuan, People's Republic of China, **2012**.
45. Liu, G.; Lin, P. Exploration and Comparison of the Essential Pathogenesis of Functional Dyspepsia Using Element and Organ Syndrome Differentiation. Proc. 24th Natl. Conf. Spleen Stomach Dis. Chin. Assoc. Tradit. Chin. Med. **2012**, 197-8.

46. Bi, W. Syndrome Differentiation Treatment of 50 Cases of Functional Dyspepsia with Tongjiang Weikang Decoction. *Chin. Med. Mod. Dist. Educ. China* **2012**, 10, 16-17.
47. Liu, H. Syndrome Differentiation Treatment of 60 Cases of Functional Dyspepsia. *Shaanxi J. Tradit. Chin. Med.* **2011**, 32, 528-529.
48. Lei, X. The Study of Syndrome Differentiation in Traditional Chinese Medicine of Functional Dyspepsia and the Relationship between LEP and PYY. MA thesis: Guangzhou Univ. Chin. Med., Guangzhou, People's Republic of China, **2011**.
49. Lu, H. Efficacy Observation of TCM Syndrome Differentiation Treatment of Spleen and Stomach Qi Deficiency Type Functional Dyspepsia. *China Foreign. Med. Treat.* **2011**, 30, 123.
50. Wang, J. TCM Syndrome Differentiation and Treatment of Functional Dyspepsia. *Gansu J. Tradit. Chin. Med.* **2010**, 23, 45-46.
51. Su, G. Current Status of Research on TCM Syndrome Differentiation Treatment of Functional Dyspepsia. *China Mod. Doctor* **2010**, 48, 13-14.
52. Hua, Z.; Cai, H. Observation on the Efficacy of Syndrome Differentiation Treatment of 45 Cases of Functional Dyspepsia. *Shandong J. Tradit. Chin. Med.* **2010**, 29, 670-671.
53. Chen, L. Syndrome Differentiation and Treatment Characteristics of Functional Dyspepsia. Proc. 22nd Natl. Symp. Integr. Tradit. Chin. West. Med. Digest. Syst. Dis. **2010**, 666-668.
54. Cao, D.; Yang, Z. Analysis of 87 Cases of Functional Dyspepsia Treated by TCM Syndrome Differentiation and Classification. *Aerospace Med.* **2010**, 21, 279-280.
55. Pan, F. Syndrome Differentiation and Classification Treatment of Functional Dyspepsia. *Mod. J. Integr. Tradit. Chin. West. Med.* **2010**, 19, 3760-3761.
56. Li, C.Y.; Wu, H.Z.; Ou, H.J.; Qiu, Q. Clinical Observation of Elderly Patient's Functional Dyspepsia Treated with Dialectic. *Liaoning J. Tradit. Chin. Med.* **2010**, 37, 2413-2414.
57. Su, G. Observation on the Efficacy of Syndrome Differentiation Treatment of 40 Cases of Functional Dyspepsia. *Nei Mongol J. Tradit. Chin. Med.* **2010**, 29, 11-12.
58. Zhang, F. Analyzing TCM Syndromes of Functional Dyspepsia and the Relationship between Various Involving Factors. MA thesis: Chengdu Univ. Trad. Chin. Med., Chengdu, People's Republic of China, **2010**.
59. Guo, X. Distribution Rule of Syndrome Differentiation in Traditional Chinese Medicine in Functional Dyspepsia. MA thesis: Guangzhou Univ. Chin. Med., Guangzhou, People's Republic of China, **2010**.
60. Zeng, C. Thoughts on TCM Syndrome Differentiation and Treatment of Functional Dyspepsia. *Chin. J. Ethnomed. Ethnopharm.* **2010**, 19, 122-123.
61. Wei, W.; Shi, H.X.; Fan, L.N. The relation between functional dyspepsia's Rome Ⅲ criteria and traditional Chinese medicine syndrome. *Glob. Tradit. Chin. Med.* **2009**, 2, 253-258.
62. Zhang, B. Efficacy Observation of TCM Syndrome Differentiation Treatment of 54 Cases of Spleen and Stomach Qi Deficiency Type Functional Dyspepsia. *Heilongjiang J. Tradit. Chin. Med.* **2009**, 38, 10-12.
63. Zheng, Z. Summary of Experience in TCM Syndrome Differentiation Treatment of Functional Dyspepsia. *Chin. J. Ethnomed. Ethnopharm.* **2009**, 18, 74-75.
64. Cai, F. Efficacy Observation of TCM Syndrome Differentiation Treatment of 120 Cases of Spleen and Stomach Qi Deficiency Type Functional Dyspepsia. *Chin. J. Mod. Drug. Appl.* **2009**, 3, 115-116.
65. Xue, L.; Zhang. H. Syndrome Differentiation and Classification Treatment of 60 Cases of Functional Dyspepsia. *J. Liaoning Univ. Tradit. Chin. Med.* **2008**, 2, 99-100.
66. Guan, S. Analysis of Syndrome Differentiation and Treatment of Functional Dyspepsia. *J. Med. Forum* **2008**, 1, 71-72.
67. Li, Y.; Wei, Y. TCM Syndrome Differentiation Treatment of Functional Dyspepsia. *Chin. Med. Mod. Dist. Educ. China* **2008**, 1, 66.

68. Liu, S. Syndrome Differentiation and Treatment of Functional Dyspepsia. *Public Med. Forum Mag.* **2008**, 14, 439-440.
69. Zhao, L. TCM Syndrome Differentiation Treatment of Functional Dyspepsia. *Guangming J. Chin. Med.* **2007**, 12, 20-21.
70. Zhang, S. Syndrome Differentiation and Classification Treatment of 32 Cases of Refractory Functional Dyspepsia. *J. Pract. Med.* **2007**, 12, 1936.
71. Xi, D. Clinical Observation of 52 Cases of Functional Dyspepsia Treated by Syndrome Differentiation. *Forum Tradit. Chin. Med.* **2007**, 5, 22-23.
72. Zhao, X. Syndrome Differentiation and Classification Treatment of 40 Cases of Functional Dyspepsia. *Shaanxi J. Tradit. Chin. Med.* **2006**, 9, 1051-1052.
73. Li, G. Syndrome Differentiation and Treatment of 76 Cases of Functional Dyspepsia. *Tradit. Chin. Med. Res.* **2006**, 6, 40-41.
74. Fan, H. Summary of 45 Cases of Functional Dyspepsia Treated by Syndrome Differentiation and Classification. *J. Sichuan Tradit. Chin. Med.* **2006**, 3, 52-53.
75. Wang, Y. Syndrome Differentiation and Classification Treatment of 86 Cases of Functional Dyspepsia. *J. Sichuan Tradit. Chin. Med.* **2006**, 6, 49-50.
76. Liu, S. TCM Syndrome Differentiation and Treatment of Functional Dyspepsia. *J. Pract. Med. Tech.* **2006**, 23, 4241-4242.
77. Gu, C.; Zhang, L.; Li, W. Clinical Research on Syndrome Differentiation and Classification Treatment of Functional Dyspepsia. *Chin. Arch. Tradit. Chin. Med.* **2006**, 5, 959-960.
78. Ye, P. Observation on the Efficacy of Syndrome Differentiation Treatment of Functional Dyspepsia. *Chin. J. Rural Med. Pharm.* **2006**, 8, 53.
79. Wang, J.; Zhou, W. Analysis of 51 Cases of Functional Dyspepsia Treated by Syndrome Differentiation. *J. Pract. Tradit. Chin. Intern. Med.* **2005**, 3, 226-267.
80. An, S. Analysis of 80 Cases of Functional Dyspepsia Treated by Syndrome Differentiation. *J. Pract. Tradit. Chin. Intern. Med.* **2005**, 3, 234.

Supplement S2. Template of the final version of the standard pattern identification tool for functional dyspepsia.

The Standard Tool of Pattern Identification for Functional Dyspepsia

Please read each question regarding your symptoms carefully, and select the score that most closely reflects your average condition over the last 2 weeks. Mark the appropriate box based on your symptoms with a checkmark (✓).

Categories	Symptoms	Never	Almost never	Sometimes	Often	Always
1. Abdomen	1. My upper abdomen feels tight and occasionally mildly painful.	☐0	☐1	☐2	☐3	☐4
	2. My upper abdomen feels tight and occasionally severely painful.	☐0	☐1	☐2	☐3	☐4
	3. My upper abdomen feels bloated and occasionally painful.	☐0	☐1	☐2	☐3	☐4
	4. My upper abdomen feels heavy and painful, like indigestion, and the pain worsens when pressed.	☐0	☐1	☐2	☐3	☐4
	5. My chest and upper abdomen feel sore or painful or hungry, making me uncomfortable.	☐0	☐1	☐2	☐3	☐4
	6. Acid reflux makes my stomach feel sore.	☐0	☐1	☐2	☐3	☐4
	7. My stomach feels bloated and gurgles.	☐0	☐1	☐2	☐3	☐4
2. Chest and Hypochondrium	1. My chest feels tight.	☐0	☐1	☐2	☐3	☐4
	2. My flank feels bloated or painful.	☐0	☐1	☐2	☐3	☐4

3. Mouth	1. I have no appetite, but my mouth is not dry.	☐0	☐1	☐2	☐3	☐4
	2. My mouth is dry or bitter.	☐0	☐1	☐2	☐3	☐4
	3. My mouth is dry or bitter, but I do not feel like drinking water.	☐0	☐1	☐2	☐3	☐4
4. Nausea and Vomiting	1. I sometimes feel nauseous or vomit.	☐0	☐1	☐2	☐3	☐4
	2. I sometimes feel nauseous or vomit, and the symptoms reduce after vomiting.	☐0	☐1	☐2	☐3	☐4
	3. I have vomited undigested food.	☐0	☐1	☐2	☐3	☐4
	4. Acid frequently regurgitates into my mouth.	☐0	☐1	☐2	☐3	☐4
5. Food	1. I eat small amounts and feel full easily.	☐0	☐1	☐2	☐3	☐4
	2. I do not feel like eating.	☐0	☐1	☐2	☐3	☐4
	3. I have no appetite and feel tightness after eating.	☐0	☐1	☐2	☐3	☐4
6. Belching	1. I belch frequently.	☐0	☐1	☐2	☐3	☐4
	2. I belch and hiccup frequently.	☐0	☐1	☐2	☐3	☐4
	3. Belching produces a foul smell and acid regurgitation.	☐0	☐1	☐2	☐3	☐4
7. Sighing	1. I sigh often.	☐0	☐1	☐2	☐3	☐4
8. Complexion	1. My face lacks luster or color.	☐0	☐1	☐2	☐3	☐4

	2. My complexion is pale and sometimes turns yellow.	☐0	☐1	☐2	☐3	☐4
9. Mental State	1. I feel mentally exhausted and my whole body is weak.	☐0	☐1	☐2	☐3	☐4
	2. I feel heat and tightness in my chest, occasionally accompanied by a feverish sensation throughout my body.	☐0	☐1	☐2	☐3	☐4
	3. My chest feels hot and tight, and I get angry easily.	☐0	☐1	☐2	☐3	☐4
10. Body and Extremities	1. My body feels heavy and lethargic.	☐0	☐1	☐2	☐3	☐4
	2. My limbs feel weak, and my hands and feet are cold.	☐0	☐1	☐2	☐3	☐4
11. Cold and Heat	1. My pain decreases when my abdomen is warmed or massaged.	☐0	☐1	☐2	☐3	☐4
12. Urination and Bowel Movements	1. My urine is dark and scanty.	☐0	☐1	☐2	☐3	☐4
	2. My stools are loose.	☐0	☐1	☐2	☐3	☐4
	3. I have difficulty with bowel movements or experience diarrhea.	☐0	☐1	☐2	☐3	☐4
	4. My stools are hard or loose, and I do not feel relieved after a bowel movement.	☐0	☐1	☐2	☐3	☐4
	5. My flatulence smells bad.	☐0	☐1	☐2	☐3	☐4

Supplement S3. Final version of score-calculation formula for each pattern identification item.

Items	Score-calculation formula
SSDC	(1–1 score*10.72)+(3–1 score*11.14)+(5–1 score*15.65)+(8–2 score*11.98)+(9–1 score*12.66)+(10–2 score*17.36)+(11–1 score*11.71)+(12–2 score *8.78)
SDQS	(1–3 score*11.29)+(2–1 score*13.19)+(5–3 score*16.48)+(6–2 score*12.45)+(8–1 score*14.28)+(9–1 score*10.71)+(9–3 score*9.15)+(12–2 score*12.45)
LSDH	(1–3 score*9.01)+(1–5 score*8.28)+(1–6 score*9.05)+(2–1 score*12.13)+(2–2 score*12.66)+(3–2 score*6.97)+(4–1 score*9.09)+(5–2 score*8.45)+(6–2 score*8.07)+(7–1 score*8.12)+(9–3 score*8.16)
TACH	(1–2 score*7.36)+(1–5 score*11.01)+(1–6 score*8.25)+(1–7 score*13.59)+(3–2 score*12.99)+(4–4 score*9.17)+(5–2 score*14.44)+(9–2 score*12.81)+(12–2 score*10.38)
DHSS	(1–2 score*13.85)+(1–5 score*10.18)+(1–6 score*8.51)+(3–3 score*10.80)+(4–1 score*7.79)+(6–1 score*12.68)+(10–1 score*7.80)+(12–1 score*12.39)+(12–4 score*16.02)
FRDO	(1–4 score*31.27)+(4–2 score*14.53)+(4–3 score*9.14)+(5–2 score*13.39)+(6–3 score*12.36)+(12–3 score*10.02)+(12–5 score*9.27)

Abbreviations. DHSS, dampness and heat in the spleen and stomach; FRDO, food retention disorder; LSDH, liver-stomach disharmony; SDQS, spleen deficiency with qi stagnation; SSDC, spleen and stomach deficiency and cold; TACH, tangled cold and heat.
